# P300/HDAC1 regulates the acetylation/deacetylation and autophagic activities of LC3/Atg8–PE ubiquitin-like system

**DOI:** 10.1038/s41420-021-00513-0

**Published:** 2021-05-31

**Authors:** Wenmei Wu, Kang Li, Sanyou Guo, Jing Xu, Qiuqin Ma, Shuyan Li, Xianying Xu, Zhijun Huang, Yangjin Zhong, Gianluca Tettamanti, Yang Cao, Sheng Li, Ling Tian

**Affiliations:** 1 Guangdong Laboratory for Lingnan Modern Agriculture/Guangdong Provincial Key Laboratory of Agro-animal Genomics and Molecular Breeding, College of Animal Science, South China Agricultural University, 510642 Guangzhou, China; 2 Guangdong Provincial Sericulture and Mulberry Engineering Research Center, College of Animal Science, South China Agricultural University, 510642 Guangzhou, China; 3 Guangdong Provincial Key Laboratory of Insect Developmental Biology and Applied Technology, Institute of Insect Science and Technology, School of Life Sciences, South China Normal University, 510631 Guangzhou, China; 4 Department of Biotechnology and Life Sciences, University of Insubria, 21100 Varese, Italy; 5 BAT Center–Interuniversity Center for Studies on Bioinspired Agro-Environmental Technology, University of Napoli Federico II, 80138 Napoli, Italy; 6 State Key Laboratory of Silkworm Genome Biology, Southwest University, 400716 Chongqing, China; 7 Biological Science Research Center/Chongqing Engineering and Technology Research Center for Novel Silk Materials, Southwest University, 400716 Chongqing, China

**Keywords:** Acetylation, Macroautophagy

## Abstract

Protein acetylation plays potential roles in regulating autophagy occurrence. However, it varies greatly between yeast and mammals, and has not been thoroughly investigated in other organisms. Here, we reported that the components of BmAtg8–PE ubiquitin-like system (BmAtg3, BmAtg4, BmAtg7, and BmAtg8) in *Bombyx mori* were localized in the nucleus under nutrient-rich conditions, whereas they were exported to the cytoplasm upon autophagy induction. RNAi of *BmP300* and inhibition of BmP300 activity resulted in nucleo-cytoplasmic translocation of BmAtg3 and BmAtg8, as well as premature induction of autophagy in the absence of stimulus. Conversely, RNAi of BmHDAC1 and inhibition of class I/II HADCs activities led to the nuclear accumulation of BmAtg3 and BmAtg8. In addition, acetylation sites in Atg proteins of BmAtg8–PE ubiquitin-like system were identified by mass spectrometry, and acetylation-site mutations caused nucleo-cytoplasmic translocation of BmAtg3, BmAtg4, and BmAtg8 along with autophagy promotion. Similarly, the subcellular localization of human ATG4b is determined by acetylation modification. In general, BmP300-mediated acetylation sequesters the components of BmAtg8–PE ubiquitin-like system in the nucleus, thus leading to the autophagy inhibition. Oppositely, BmHDAC1-mediated deacetylation leads to the nucleo-cytoplasmic translocation of the components of BmAtg8–PE ubiquitin-like system and promotes autophagy. This process is evolutionarily conserved between insects and mammals.

## Introduction

Macroautophagy/autophagy, which is implicated in neurodegenerative diseases, tumorigenesis, and pathogen invasion, is a finely regulated cellular process for bulk degradation of cellular components^1^. Autophagosome biogenesis requires the participation of a series of autophagy-related (Atg) proteins, and the maturation of autophagosome involves in the ubiquitin-like systems, LC3/Atg8–phosphatidylethanolamine (PE)^1^. LC3/Atg8–PE conjugation is mainly catalyzed by Atg4, Atg7, and Atg3 in a sequential manner^1,2^. Atg4 is the sole cysteine protease responsible for the cleavage of nascent LC3/Atg8^3^. After exposure of the C-terminal, LC3/Atg8 is adenylated by the E1-like enzyme Atg7 to form a covalent thioester-linked intermediate, and then is transferred to E2-like enzyme Atg3 before conjugation with PE^2,4,5^. There are four *Atg4* genes in mammals, one in yeast, two in *Caenorhabditis elegans* and *Drosophila melanogaster*, respectively^2,6^. The protease activity of different ATG4 to different LC3 homologs is notably varied^7,8^. Mammalian ATG4b cleaves most LC3 homologs, and is considered as a potential biomarker and therapeutic target in human diseases^9,10^. Atg7 and Atg3 homologs are associated with the control of autophagosome size and number, and act as the indicators of adverse disease prognosis and anticancer targets in mammals^11–13^. Several catalytic components of LC3/Atg8–PE conjugation system have been functionally identified in invertebrates; however, the molecular mechanism of regulation by acetylation remains largely unknown^10,14,15^.

Autophagy is strictly regulated by nutrients and energy signaling mainly through the phosphorylation of ULK1/Atg1-ATG13/Atg13 protein complex^16,17^. Moreover, acetylation of lysine sites in Atg proteins represents a universal mechanism involved in the regulation of autophagy^18,19^. Starvation rapidly depletes acetyl-coenzyme A, which subsequently downregulates the activity of histone acetyltransferase hMOF/KAT8/MYST1 and results in the deacetylation of histone H4 at lysine16 (H4K16ac), thus leading to autophagy induction^20–22^. In *Drosophila*, knockdown of *AcCoAS/acetyl-coenzyme A synthetase* in the brain enhances autophagy and lifespan, indicating a regulation of autophagy by acetylation both in insects and mammals^22,23^. Whereas, it is worthy to note that yeast Atg3 is hyperacetylated during starvation-induced autophagy^24^. Yeast acetyltransferase ESA1-dependent acetylation facilitates Atg3 to participate in autophagy, in comparison, deletion of deacetylase *Rpd3* increases autophagy^24^. In mammals, deacetylation of ATG proteins, including ATG5, ATG7, LC3, and ATG12, by sirtuin 1 (SIRT1), histone deacetylase 6 (HDAC6), or HDAC1 promotes, while acetylase EP300/P300-mediated acetylation inhibits, autophagy^18,25–28^. Accordingly, EP300/P300 sequesters LC3 in the nucleus under nutrient-rich conditions, while deacetylation of LC3, catalyzed by the deacetylase SIRT1, results in nucleo-cytoplasmic translocation of LC3 and consequent autophagy promotion under starvation^19,29^. Up to now, the variation of regulation by acetylation in autophagy through the modification of LC3/Atg8–PE ubiquitin-like system observed in mammals and yeast is not documented in other species^19,24^.

In insects, 20-hydroxyecdysone (20E), the steroid hormone synthesized from dietary cholesterol, is a key regulator of multiple processes during molting and metamorphosis^30,31^. During larval–pupal transition, 20E markedly induces autophagy by upregulating the expression of *Atg* genes and simultaneously inhibiting MTORC1 (mechanistic target of rapamycin kinase complex 1) activity in both *D. melanogaster* and *Bombyx mori*^30,32,33^. Our previous study has shown that cholesterol and its derivatives can induce the dephosphorylation of histone deacetylase BmRpd3/HsHDAC1, and determine their nucleo-cytoplasmic translocation to promote autophagy both in *B. mori* and humans^34^, suggesting an involvement of deacetylation in autophagy regulation. However, the precise regulatory mechanism of BmHDAC1 (BmRpd3) on autophagy still needs to be determined. In order to investigate the discrepancy in acetylation regulation of LC3/Atg8–PE ubiquitin-like system components in organisms other than yeast and mammals, here we investigated this regulatory mechanism in *B. mori*, an organism of intermediate evolutionary status. Our results demonstrate that the regulation of autophagy by acetylation, through the modification of the components of Atg8–PE ubiquitin-like system in *B. mori*, is evolutionarily conserved with mammals, while is opposite to that of yeast.

## Results

### BmAtg3 and BmAtg8 are required for autophagy occurrence during larval–pupal metamorphosis

**Fig. 1:**
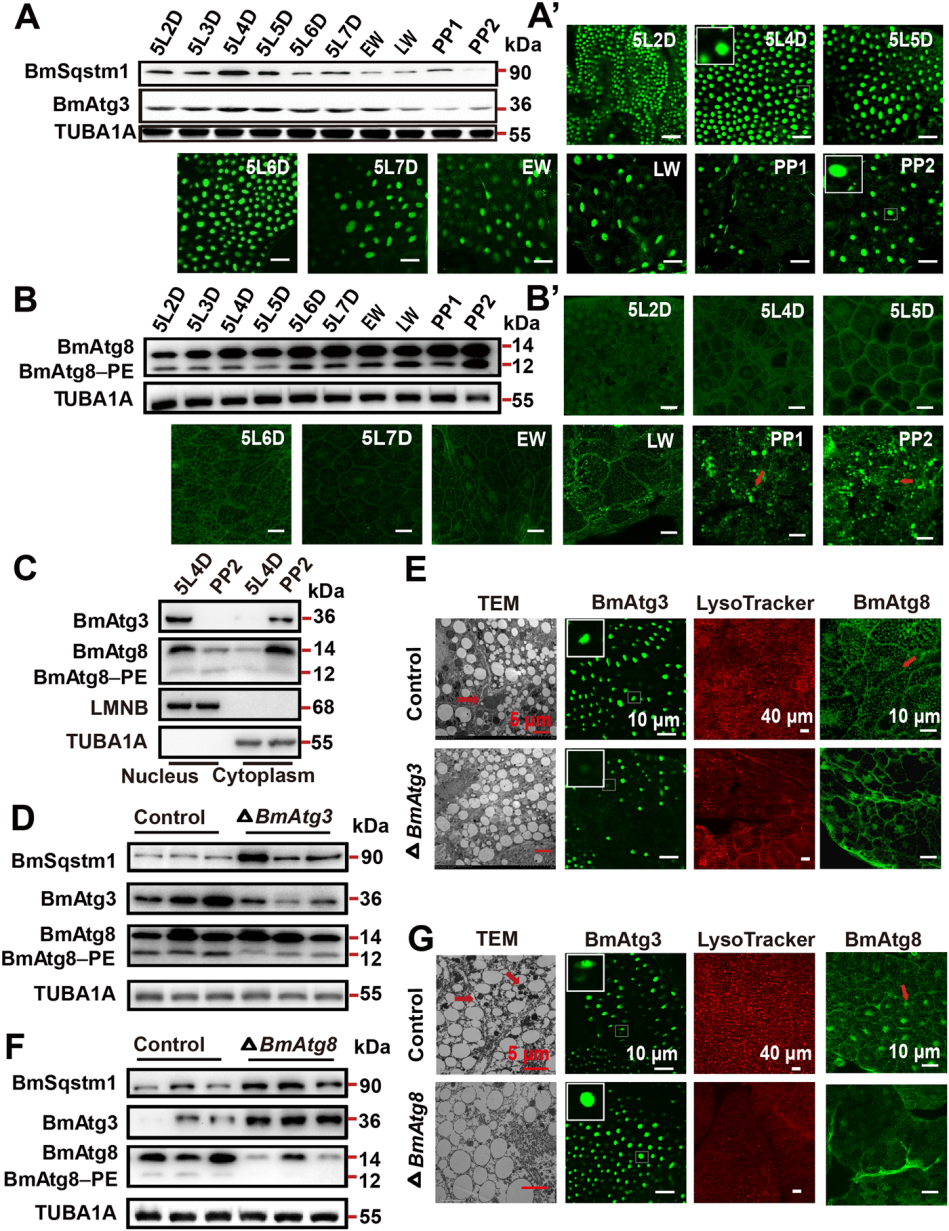
BmAtg8 and BmAtg3 are essential for autophagy induction in *B. mori* fat body. **A**–A′ Protein levels of BmSqstm1 and BmAtg3 (**A**), immunofluorescent staining of BmAtg3 (A′) in *B. mori* fat body from 5L2D to PP2 stage. EW early wandering stage, LW later wandering stage. Scale bar: 10 µm. **B**–B′ Protein levels (**B**) and immunofluorescent staining (B′) of BmAtg8 in *B. mori* fat body from 5L2D to PP2 stage. Arrows: typical fat body cell. Scale bar: 10 µm. **C** Protein levels of BmAtg3 and BmAtg8 in the nucleus and cytoplasm at 5L4D and PP2 stages. **D** Protein levels of BmSqstm1, BmAtg3, and BmAtg8 after *BmAtg3* knockout. **E** LysoTracker Red staining, TEM analysis, and immunofluorescent staining of BmAtg3 and BmAtg8 after *BmAtg3* knockout. **F** Protein levels of BmSqstm1, BmAtg3, and BmAtg8 after *BmAtg8* knockout. **G** LysoTracker Red staining, TEM analysis, and immunofluorescent staining of BmAtg3 and BmAtg8 after *BmAtg8* knockout.

To investigate the autophagic function of BmAtg8–PE ubiquitin-like system, *BmAtg3* (NP_001135961.1), *BmAtg8* (NM_001046779.1), *BmAtg4* (XM_021346895), and *BmAtg7* (KY608887.1) were identified in *B. mori*. For the transcriptional levels of *BmAtg3* and *BmAtg8* are fairly high during autophagy occurrence^30^, their protein levels and subcellular localization were analyzed in the fat body from 5L2D to PP2. Western blotting and immunofluorescent staining showed that the protein level of BmAtg3 gradually reduced from late fifth instar to prepupal stage, similar to the variation of BmSqstm1, the selective autophagy receptor/adaptor. Interestingly, BmAtg3 located in the nucleus at the feeding stage, but weak BmAtg3 staining was observed in the cytoplasm at the prepupal stage (Fig. 1A–A′ and Supplementary Fig. S1A). The protein level of BmAtg8–PE gradually increased from 5L2D to PP2. BmAtg8 located in both nucleus and cytoplasm at the feeding stage, while BmAtg8-positive puncta were clearly visible in the cytoplasm at the prepupal stage when autophagy occurred (Fig. 1B–B′ and Supplementary Fig. S1B). We then separated nuclear proteins from cytoplasmic proteins, and western blotting showed that the cytoplasmic distribution of BmAtg3 and BmAtg8 significantly enhanced at PP2 compared to 5L4D (Fig. 1C). In summary, BmAtg3 and BmAtg8 are exported from the nucleus to cytoplasm when autophagy occurs.

The autophagic function of *BmAtg3* and *BmAtg8* was investigated via CRISPR/Cas9-mediated knockout experiments. The efficiency of *BmAtg3* and *BmAtg8* knockouts was ~30% and ~40%, respectively (Supplementary Fig. S1C, D). The BmAtg3 protein level in the fat body was significantly diminished by *BmAtg3* knockout; meanwhile, BmAtg8–PE conjugation was reduced and BmSqstm1 protein level was accumulated during the autophagy induction (Fig. 1D). Transmission electron microscopy (TEM) analysis, LysoTracker staining, and immunofluorescent staining confirmed the decrease in autophagosome and autolysosome formation, notably, BmAtg8 was accumulated in the nucleus after *BmAtg3* knockout (Fig. 1E). *BmAtg8* knockout reduced both BmAtg8 and BmAtg8–PE protein, as well as autophagy monitored by TEM analysis and LysoTracker staining, moreover, BmAtg3 was accumulated in the nucleus (Fig. 1F, G). These results demonstrate that both *BmAtg3* and *BmAtg8* are required for autophagy, and exhibit nucleo-cytoplasmic translocation during autophagy induction.

### BmAtg3 and BmAtg8 are exported from the nucleus to cytoplasm during 20E/starvation-induced autophagy

**Fig. 2:**
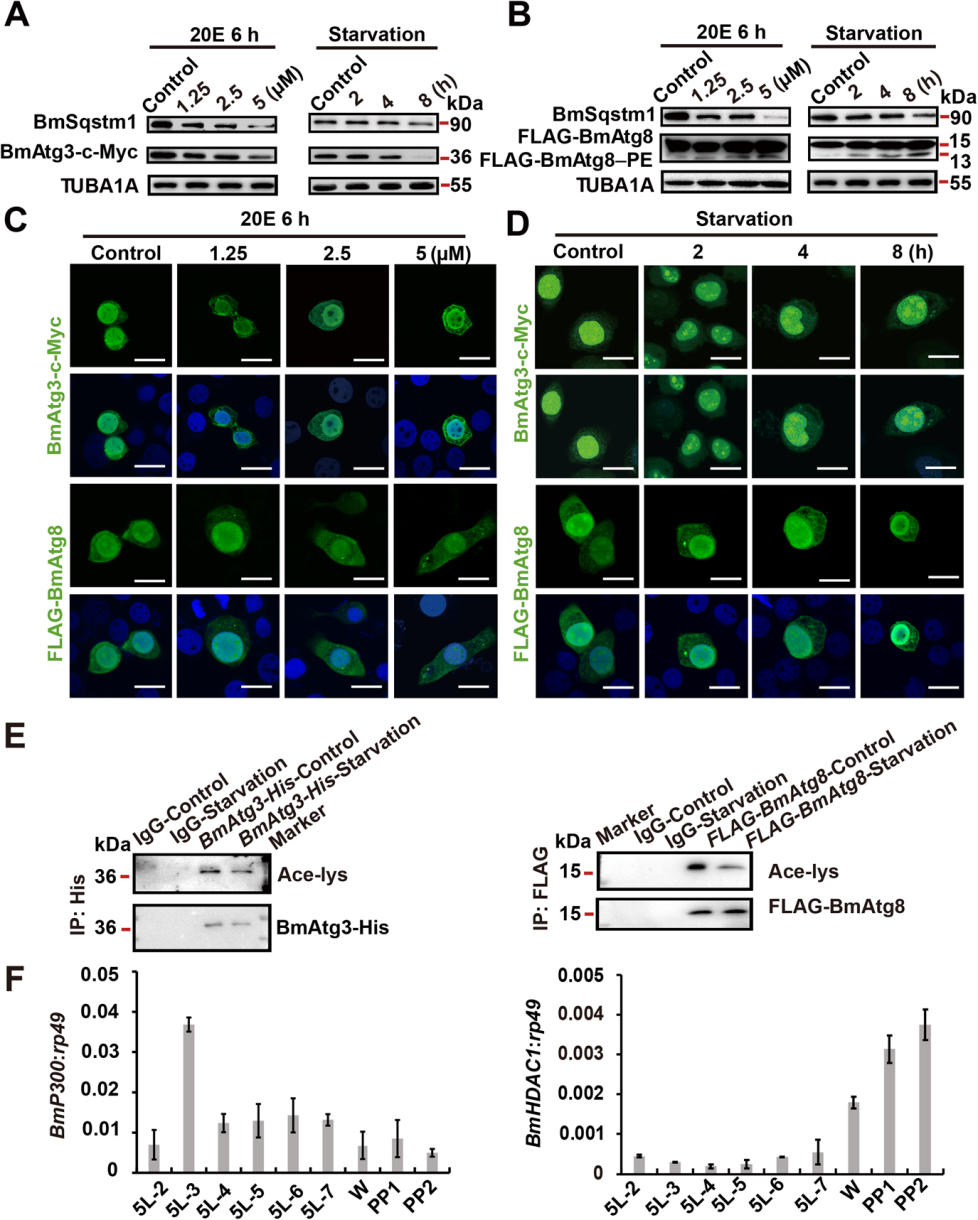
Nuclear export of BmAtg3 and BmAtg8 is associated with autophagy induction. **A** Protein levels of BmSqstm1 and BmAtg3-c-Myc in *BmAtg3-c-Myc-*overexpressing BmN cells treated with 1, 2.5, and 5 μM 20E for 6 h or 2, 4, and 8 h starvation. **B** Protein levels of BmSqstm1 and FLAG-BmAtg8 in *FLAG-BmAtg8-*overexpressing BmN cells treated with 1, 2.5, and 5 μM 20E for 6 h or 2, 4, and 8 h starvation. **C** Immunofluorescent staining of BmAtg3-c-Myc and FLAG-BmAtg8 after 1, 2.5, and 5 μM 20E treatments for 6 h in BmN cells. Scale bar: 10 µm. **D** Immunofluorescent staining of BmAtg3-c-Myc and FLAG-BmAtg8 after 2, 4, or 8 h starvation in BmN cells. Scale bar: 10 µm. **E** Acetylation levels of BmAtg3 and BmAtg8 after starvation for 4 h. **F** Transcriptional levels of acetyltransferases *BmP300* and deacetylase *BmHDAC1* in *B. mori* fat body from 5L2D to PP2 stage.

20E and starvation are two most important inducers of autophagy in insects^15^^,30^^,^^35^. After treatments with different doses of 20E (1.25, 2.5, and 5 μM) or different time intervals of starvation (2, 4, and 8 h), BmSqstm1 and BmAtg3-c-Myc protein levels gradually decreased, while FLAG-BmAtg8–PE increased in BmN cells, showing a consistent variation of BmAtg3 and BmAtg8–PE formation in 20E- and starvation-induced autophagy (Fig. 2A, B). In addition, BmAtg3-c-Myc and FLAG-BmAtg8 were exported from the nucleus to cytoplasm in response to 20E or starvation, and puncta for both BmAtg3 and BmAtg8 were visible in the cytoplasm (Fig. 2C, D and Supplementary Fig. S1E, F). Similarly, both 20E and starvation treatments induced BmAtg8–PE conjugation, the decrease of BmAtg3 protein level, and their protein nuclear export, along with BmSqstm1 degradation in the fat body (Supplementary Fig. S2A, B). These results indicate that BmAtg3 and BmAtg8 undergo nucleo-cytoplasmic translocation during 20E- or starvation-induced autophagy.

### BmP300 and BmHDAC1 oppositely regulate the acetylation/deacetylation and nuclear localization of BmAtg3 and BmAtg8

Deacetylase BmHDAC1 facilitates autophagy in *B. mori*^34^, thus, whether BmAtg3 and BmAtg8 were regulated by acetylation was investigated. Results showed that the immunoprecipitated BmAtg3-His and FLAG-BmAtg8 were both acetylated under nutrient-rich conditions, while deacetylated after the starvation treatment (Fig. 2E). To analyze the regulatory machinery of acetylation in the silkworm, the developmental profiles of acetyltransferases, including *BmP300* (XP_021204790.1), *BmTip60* (XP_004928298.1), *BmCbp* (XP_021206531.1), and *BmKat2a* (XP_004922629.1), as well as deacetylases *BmSirt2* (NP_001036937.1), *BmHDAC3* (XP_012552478.1), *BmHDAC8* (XP_021205467.1), and *BmHDAC1* (XP_004931440.2) in the fat body were evaluated from 5L2D to PP2 stages. Detection showed that the mRNA level of *BmP300*, an acetylase displaying high homology with mammalian P300, was high during the feeding stage (5L3D) but low during the prepupal stage, showing the opposite trend to autophagy induction (Fig. 2F and Supplementary Fig. S2C). In comparison, the expression of *BmHDAC1* was low during the feeding stage, but steadily increased during larval–pupal transition, indicating the consistency with autophagy occurrence (Fig. 2F and Supplementary Fig. S2C).

**Fig. 3:**
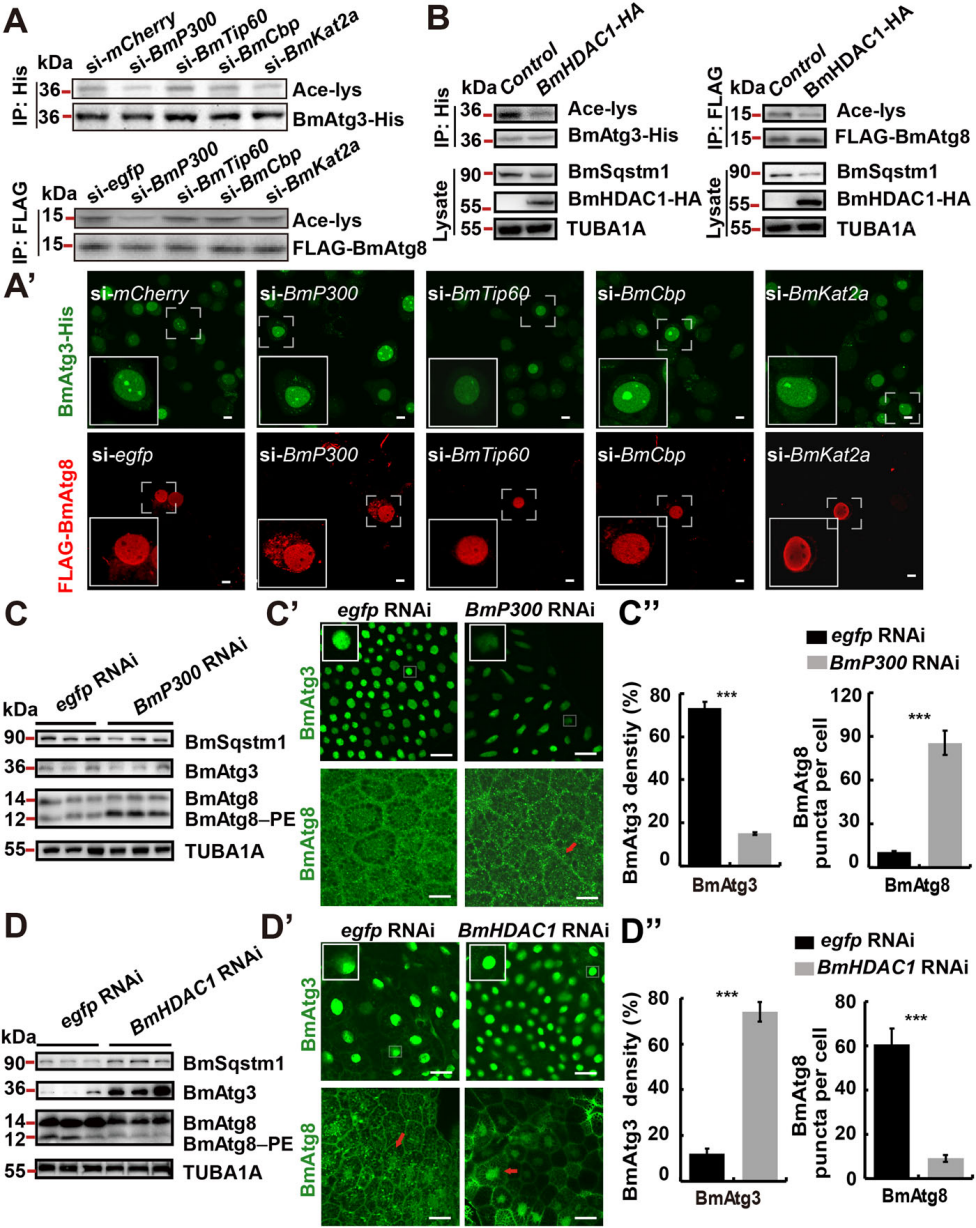
*BmP300* and *BmHDAC1* oppositely regulate acetylation and nuclear localization of BmAtg3 and BmAtg8. **A**–A′ Acetylation levels of BmAtg3-His and FLAG-BmAtg8 (**A**) and immunofluorescent staining of BmAtg3 and BmAtg8 (A′) after *BmP300*, *BmTip60*, *BmCBP*, or *BmKat2a* siRNA treatment for 24 h in BmN cells. Scale bar: 10 µm. **B** Protein levels of acetylated BmAtg3-His and FLAG-BmAtg8, and BmSqstm1 after co-overexpression of *BmHDAC1* and *BmAtg3-His*/*FLAG-BmAtg8*. **C**–C″ Protein levels of BmAtg3, BmAtg8, and BmSqstm1 (**C**), immunofluorescent staining of BmAtg3 and BmAtg8 after *BmP300* RNAi treatment for 24 h in the fat body (C′). Quantification of fluorescent BmAtg3 and BmAtg8 puncta in C′ (C″). Arrows: BmAtg8 puncta. Scale bar: 10 µm. **D**–D″ Protein levels of BmAtg3, BmAtg8, and BmSqstm1 (**D**) immunofluorescent staining of BmAtg3 and BmAtg8 after *BmHDAC1* RNAi for 24 h in the fat body (D′). Quantification of fluorescent BmAtg3 and BmAtg8 puncta in D′ (D″). Scale bar: 10 µm.

Since the expression level of *BmP300* was almost consistent with, while *BmHDAC1* was opposite to, the nuclear localization of BmAtg3 and BmAtg8, they were assumed to be involved in the acetylation of BmAtg3 and BmAtg8. SiRNA for *BmP300*, *BmTip60*, *BmCbp*, and *BmKat2a* were co-transfected with *BmAtg3-His* or *FLAG-BmAtg8* in BmN cells. Results showed that *siBmP300* treatment significantly decreased the acetylation levels of BmAtg3-His and FLAG-BmAtg8, and also led to their nucleo-cytoplasmic translocation (Fig. 3A–A′ and Supplementary Fig. S2D, E). Conversely, overexpression of *BmHDAC1* led to the deacetylation of BmAtg3-His and FLAG-BmAtg8, as well as BmSqstm1 degradation (Fig. 3B). Summarizing, we prove that *BmP300* and *BmHDAC1* are the two key enzymes in *B. mori* to catalyze acetylation and deacetylation of BmAtg3 and BmAtg8, respectively.

### BmP300 inhibits while BmHDAC1 promotes autophagy

Since BmP300 and BmHDAC1 regulate the acetylation status and subcellular localization of BmAtg3 and BmAtg8, their activities in regulating autophagy were further investigated. Knockdown of *BmP300* and *BmHDAC1* was performed in vivo using RNAi at 5L3D and 12 h before IW, respectively. *BmP300* RNAi for 24 h reduced its mRNA levels to ~40% compared to *egfp* RNAi (Supplementary Fig. S2F). Autophagy, monitored by the degradation of BmSqstm1 and BmAtg8–PE conjugation, was markedly induced, meanwhile, the BmAtg3 and BmAtg8 nuclear localization were significantly decreased along with increased BmAtg8 puncta in the cytoplasm of fat body cells after *BmP300* RNAi (Fig. 3C–C″). *BmHDAC1* mRNA levels were reduced to ~50% after RNAi, consequently, the decrease of BmSqstm1 and BmAtg3, as well as BmAtg8–PE conjugation were blocked. Moreover, BmAtg3 and BmAtg8 were arrested in the nucleus along with decreased BmAtg8 puncta in the cytoplasm (Fig. 3D–D″ and Supplementary Fig. S2G).

**Fig. 4:**
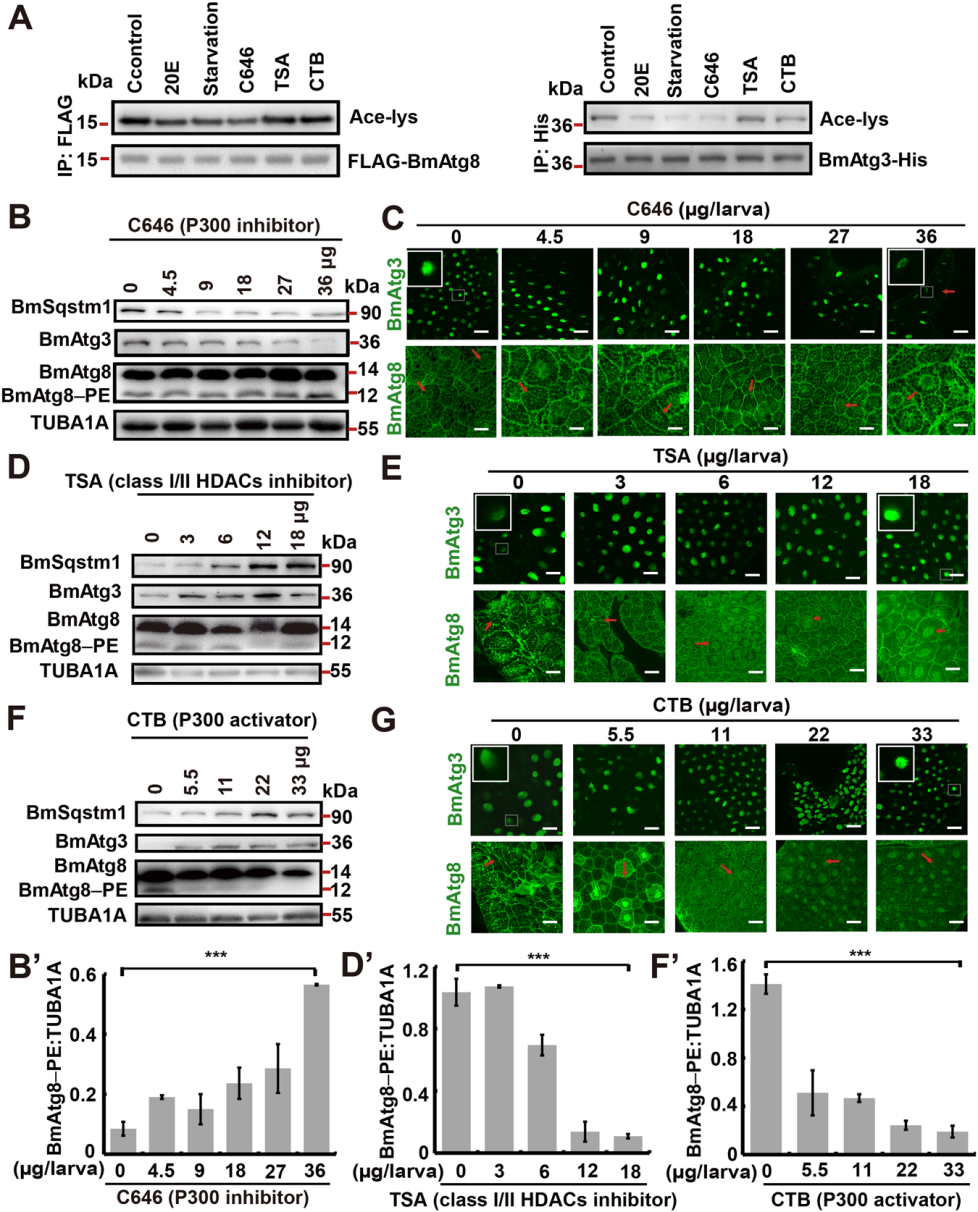
P300 and class I/II HDAC activities oppositely regulate BmAtg3 and BmAtg8 nuclear localization and autophagy. **A** Acetylation levels of BmAtg3-His and FLAG-BmAtg8 after 5 μM 20E, starvation, 800 nM C646, 20 µM TSA, or 20 µM CTB treatment for 6 h in BmN cells. IP immunoprecipitation. **B**–B′ Protein levels of BmSqstm1, BmAtg3, and BmAtg8 in the fat body after 4.5, 9, 18, 27, or 36 μg/larva C646 treatment for 24 h, 0 μg/larva treatment is used as control (**B**). Quantification of BmAtg8–PE in **B** (B′). Significance test was performed between the control and the highest dose. **C** Immunofluorescent staining of BmAtg3 and BmAtg8 in the fat body after 4.5, 9, 18, 27, or 36 μg/larva C646 treatment for 24 h. Arrows: typical treated cells. Scale bar: 10 µm. **D**–D′ Protein levels of BmSqstm1, BmAtg3, and BmAtg8 in the fat body after 3, 6, 12, or 18 μg/larva TSA treatment for 24 h (**D**). Quantification of BmAtg8–PE in **D** (D′). Significance test was performed between the control and the highest dose. **E** Immunofluorescent staining of BmAtg3 and BmAtg8 in the fat body after 3, 6, 12, or 18 μg/larva TSA treatment for 24 h. Scale bar: 10 µm. **F**–F′ Protein levels of BmAtg3, BmAtg8, and BmSqstm1 in the fat body after 5.5, 11, 22, or 33 μg/larva CTB treatment for 24 h (**F**). Quantification of BmAtg8–PE in **F** (F′). Significance test was performed between the control and the highest dose. **G** Immunofluorescent staining of BmAtg3 and BmAtg8 in the fat body after 5.5, 11, 22, or 33 μg/larva CTB treatment for 24 h. Scale bar: 10 µm.

To complement with the RNAi results, chemical compounds to modify the acetylation status in vivo were used. The effects of C646 (a selective inhibitor of mammalian P300), Trichostatin A (TSA, an effective inhibitor of deacetylase Rpd3 and its class I/II HDAC homologs in yeast and mammals), and N-(4-chloro-3-trifluoromethyl-phenyl)-2-ethoxy-benzamide (CTB, a P300/CBP specific activator) on autophagy and acetylation of BmAtg3 and BmAtg8 were analyzed. Similar to 20E and starvation treatments, C646 administration markedly decreased the acetylation levels of immunoprecipitated BmAtg3-His and FLAG-BmAtg8. The results agreed that BmP300 inhibition resulted in the deacetylation of BmAtg proteins (Fig. 4A). Moreover, treating 5L3D feeding larvae, when autophagy was rarely observed, with different doses of C646 for 24 h revealed the decrease in BmSqstm1 and BmAtg3 protein levels with the simultaneous increase in BmAtg8–PE conjugation, indicating autophagy promotion (Fig. 4B–B′). Immunofluorescent staining showed that the nuclear localization of BmAtg3 and BmAtg8 significantly decreased, while BmAtg8 cytoplasmic puncta were increased in C646-treated larvae (Fig. 4C). In contrast, different doses of TSA injection into the larvae at early wandering (EW) stage, when autophagy is initiated, resulted in BmSqstm1 and BmAtg3 accumulation, as well as inhibition of BmAtg8–PE conjugation (Fig. 4D–D′); moreover, BmAtg3 and BmAtg8 were mainly accumulated in the nucleus, and a decrease of BmAtg8 puncta in the cytoplasm of fat body cells was observed, suggesting the involvement of class I/II HDAC activities in autophagy occurrence and nucleo-cytoplasmic translocation of BmAtg3 and BmAtg8 (Fig. 4E). Similarly, the administration of different doses of CTB to larvae at EW stage inhibited the degradation of BmSqstm1 and BmAtg3, and decreased BmAtg8–PE formation, along with the nuclear accumulation of BmAtg3 and BmAtg8 (Fig. 4F–F′, G).

Similarly, the exposure of BmN cells to different doses of C646 resulted in a gradual degradation of BmSqstm1, along with an increase in BmAtg8–PE conjugation (Supplementary Fig. S3A, B). In addition, overexpressed BmAtg3 and BmAtg8 were translocated from the nucleus to cytoplasm, where they formed the puncta (Supplementary Fig. S3C). Immunofluorescent staining showed that TSA pretreatment partially blocked 20E-induced nuclear export of BmAtg3 and BmAtg8, as well as BmAtg8 punctation in the cytoplasm (Supplementary Fig. S3D). Finally, TSA or CTB pretreatment reduced 20E-induced autophagy as indicated by the decrease in BmAtg8–PE formation, as well as the accumulation of BmSqstm1, while C646 pretreatment intensively promoted BmSqstm1 degradation and BmAtg8–PE conjugation (Supplementary Fig. S3E). In summary, BmP300 and BmHDAC1 oppositely regulate the nuclear localization of BmAtg3 and BmAtg8, and thus autophagy induction in *B. mori*.

### BmAtg4 and BmAtg7 are deacetylated during autophagy induction

**Fig. 5:**
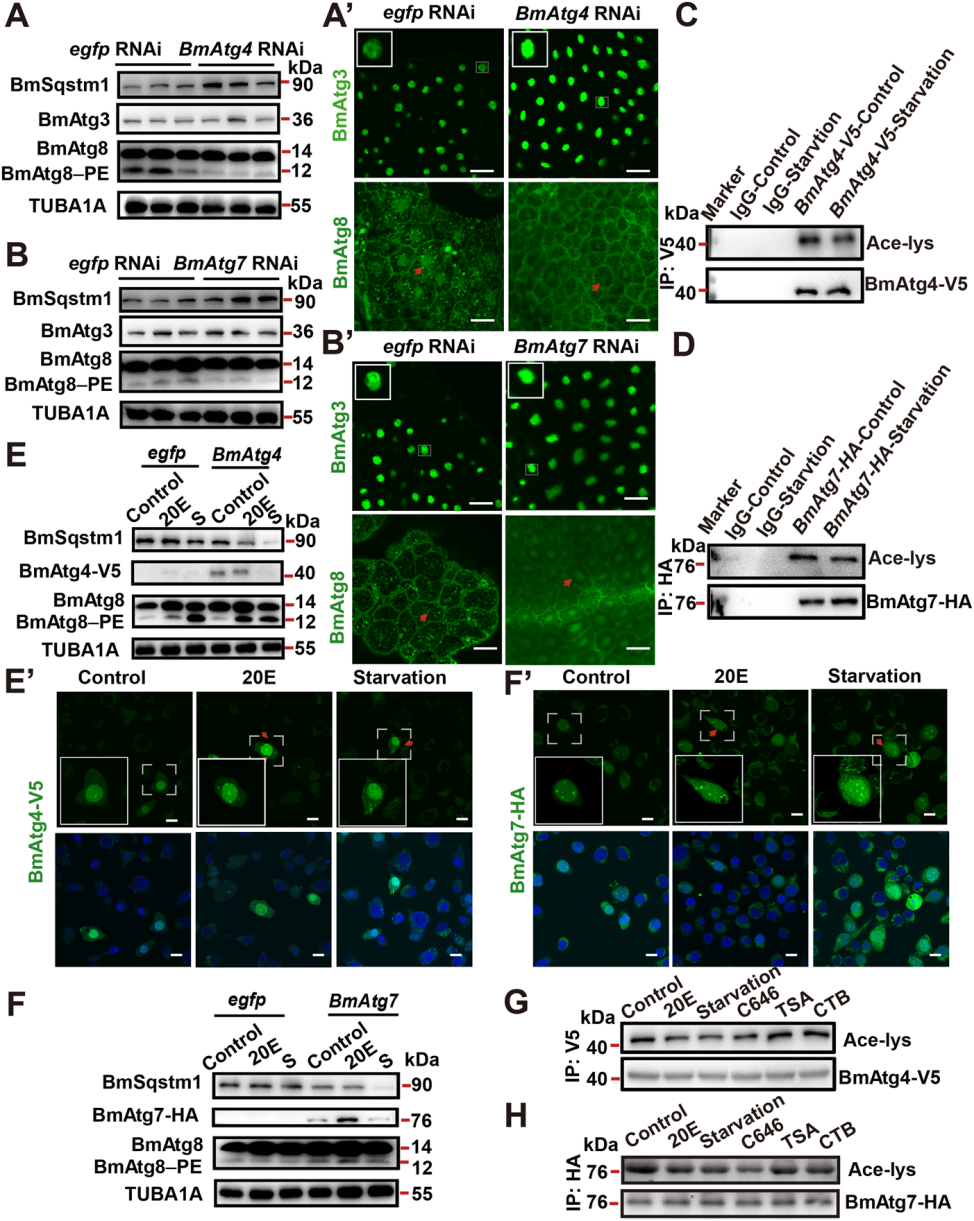
Autophagic activities of BmAtg4 and BmAtg7 are accompanied by a variation of acetylation. **A**–A′ Protein levels of BmSqstm1, BmAtg3, and BmAtg8 (**A**) and immunofluorescent staining of BmAtg3 and BmAtg8 (A’) in the fat body after *BmAtg4* RNAi for 24 h. Scale bar: 10 µm. **B**–B′ Protein levels of BmSqstm1, BmAtg3, and BmAtg8 (**B**) and immunofluorescent staining of BmAtg3 and BmAtg8 (B′) in the fat body after *BmAtg7* RNAi for 24 h. Scale bar: 10 µm. **C** Acetylation levels of BmAtg4-V5 after starvation for 4 h. **D** Acetylation levels of BmAtg7-HA after starvation for 4 h. **E**–E′ Protein levels of BmAtg8, BmSqstm1, and BmAtg4-V5 (**E**), and immunofluorescent staining of BmAtg4-V5 (E′) after 20E or starvation treatment for 4 h, S starvation. Scale bar: 10 µm. **F**–F′ Protein levels of BmSqstm1, BmAtg7-HA, and BmAtg8 (**F**), and immunofluorescent staining of BmAtg7-HA (F′) after 20E or starvation treatment for 4 h. Scale bar: 10 µm. **G** Acetylation levels of BmAtg4-V5 after 5 μM 20E, starvation, 800 nM C646, 20 µM TSA, or 20 µM CTB treatment for 6 h in BmN cells. **H** Acetylation levels of BmAtg7-HA after 5 μM 20E, starvation, 800 nM C646, 20 µM TSA, or 20 µM CTB treatment for 6 h in BmN cells.

Atg4 and Atg7 are important mediators involved in the LC3/Atg8–PE ubiquitin-like system and autophagic flux^1^^,^^12^. Nevertheless, BmAtg4 and BmAtg7 have not been functionally identified in *B. mori* yet. To investigate their roles in the silkworm, we first performed *BmAtg4* and *BmAtg7* RNAi in larvae 12 h before IW. The mRNA levels of *BmAtg4* and *BmAtg7* were reduced to ~50% and ~30% 24 h after RNAi, respectively (Supplementary Fig. S2H, I). Decrease of BmAtg8–PE conjugation and accumulation of BmSqstm1 indicated the blockage of autophagy (Fig. 5A, B). Moreover, both of BmAtg3 and BmAtg8 increased in the nuclei (Fig. 5A′, B′). These results show that BmAtg4 and BmAtg7 are required for the nuclear export of BmAtg3 and BmAtg8, and thus the autophagic activities of LC3/Atg8–PE ubiquitin-like system in *B. mori*.

We next investigated whether the subcellular localization of BmAtg4 and BmAtg7 were regulated by 20E or starvation via acetylation. Results showed that starvation-induced deacetylation of both BmAtg4-V5 and BmAtg7-HA (Fig. 5C, D). Moreover, the overexpression of *BmAtg4* or *BmAtg7* enhanced 20E- or starvation-induced autophagy, and the two Atg proteins were translocated from the nucleus to cytoplasm after 2.5 μM 20E and 4 h starvation treatments in BmN cells (Fig. 5E–E′, F–F′). Finally, the acetylation levels of BmAtg4-V5 and BmAtg7-HA were reduced by 20E, starvation, and 800 nM C646 treatments (Fig. 5G, H).

### Acetylation on lysine residues regulates subcellular localization and autophagic activities of LC3/Atg8–PE ubiquitin-like system

Since the autophagic functions of BmAtg3, BmAtg8, BmAtg7, and BmAtg4 were regulated by acetylation, their potential acetylation sites were further identified by mass spectrometry (MS). Two silkworm-specific acetylation sites at K94 and K195, which were not conserved with yeast Atg3, were identified in purified BmAtg3 (Supplementary Figs. S4A and S5A). In comparison, five acetylation sites at K6, K13, K20, K24, and K46 were identified in BmAtg8. An additional acetylation site, which was predicted to be conserved in LC3B, was found in BmAtg8 at K48 (Supplementary Figs. S4B and S5B–B′). Finally, three acetylation sites at K125, K237, and K269 in BmAtg4, and seven acetylation sites at K32, K37, K302, K311, K350, K415, and K515 in BmAtg7 were identified (Supplementary Figs. S4C, D and S5C, D).

**Fig. 6:**
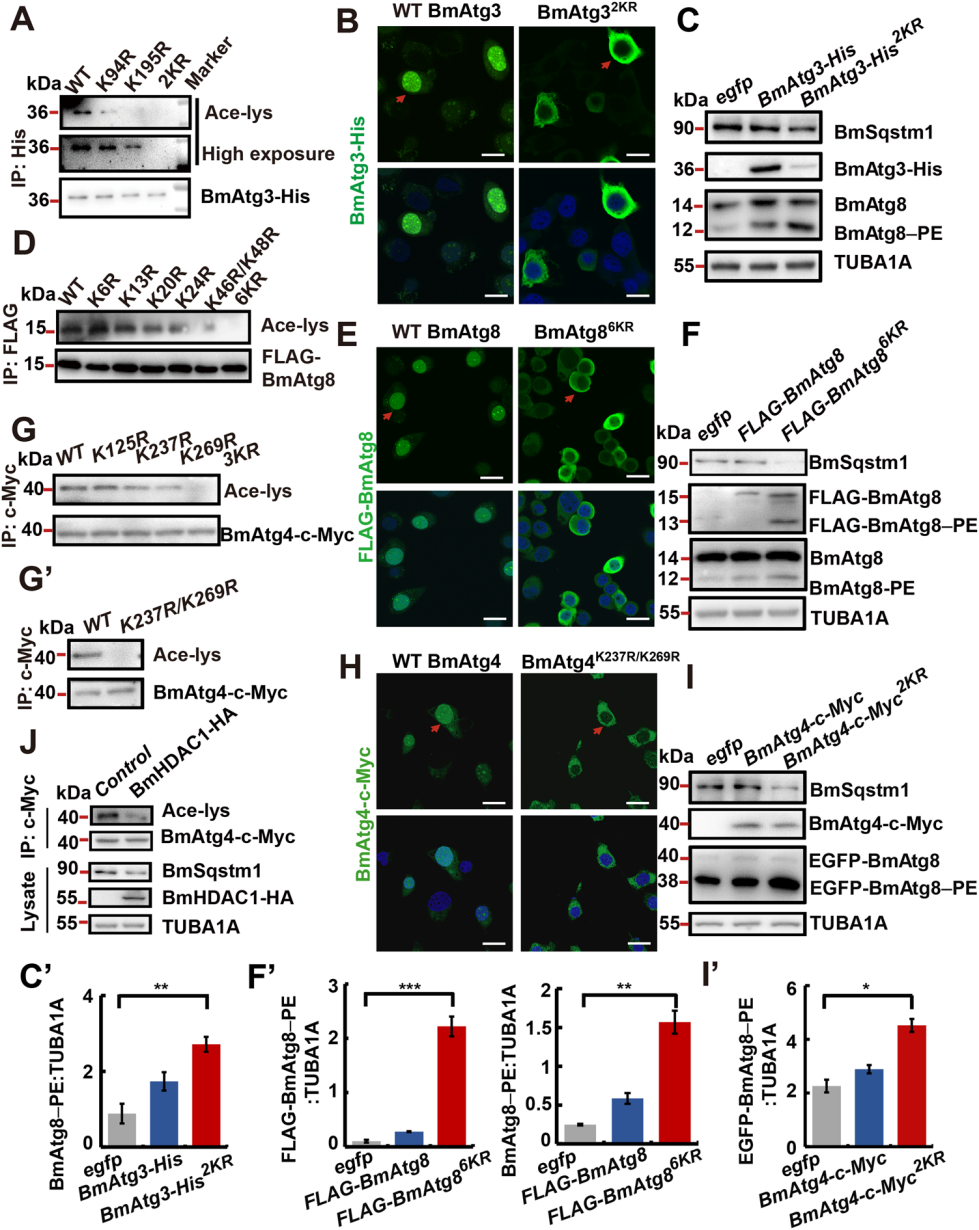
Authenticity and autophagic activities of identified acetylation sites in BmAtg3, BmAtg4, and BmAtg8. **A** Acetylation level of BmAtg3 after single and double mutation of acetylation sites. 2KR: double acetylation-site mutated from lysine (K) to arginine (R). **B** Immunofluorescent staining of BmAtg3 double acetylation-site mutant. Scale bar: 10 µm. **C**–C′ Protein levels of BmSqstm1, BmAtg8, and BmAtg3 after overexpression of double acetylation-site mutated *BmAtg3* under nutrient-rich conditions (**C**). Quantification of BmAtg8–PE in **C** (C′). Significance test was performed between *egfp* and mutant overexpression. **D** Acetylation level of BmAtg8 after single and sextuple mutation of acetylation sites. **E** Immunofluorescent staining of BmAtg8 after sextuple mutation of acetylation sites. 6KR: sextuple acetylation site mutation from lysine to arginine. Scale bar: 10 µm. **F**–F′ Protein levels of BmSqstm1, FLAG-BmAtg8, and endogenous BmAtg8 after overexpression of sextuple acetylation-site mutated *BmAtg8* under nutrient-rich conditions (**F**). Quantification of BmAtg8–PE and EGFP- BmAtg8–PE in **F** (F′). **G**–G′ Acetylation of BmAtg4 after single and triple (**G**), or double (G′) mutation of acetylation sites. **H** Immunofluorescent staining of BmAtg4 after double mutation of acetylation sites. Scale bar: 10 µm. **I**–I′ Protein levels of BmSqstm1 and EGFP-BmAtg8 after overexpression of double acetylation-site mutated *BmAtg4* under nutrient-rich conditions (**I**). Quantification of EGFP-BmAtg8–PE in **I** (I′). **J** Acetylation of c-Myc-BmAtg4, and protein levels of BmSqstm1 and BmHDAC1 after *BmHDAC1* overexpression.

Subsequently, acetylation sites in BmAtg3, BmAtg8, and BmAtg4 were verified. Results showed that single mutation partly attenuate and double mutation of acetylation sites totally abolished BmAtg3 acetylation, which consequently resulted in its cytoplasmic localization and premature autophagy induction, as indicated by BmAtg3 immunofluorescent staining, BmSqstm1 degradation, and BmAtg8–PE conjugation (Fig. 6A–C–C′). Mutation of acetylation sites at K13, K20, K24, or K46/K48 but not K6 partly decreased, while sextuple mutation of acetylation sites abolished, the acetylation of BmAtg8, which led to completely cytoplasmic localization of BmAtg8 and premature autophagy induction (Fig. 6D–F–F′). Interestingly, most of the double acetylation-site mutated BmAtg3 and sextuple acetylation-site mutated BmAtg8 were colocalized in the cytoplasm after their co-overexpression (Supplementary Fig. S6A). Since the regulatory mechanism of acetylation in Atg4 homologs has not been elucidated yet, we further analyzed the acetylation sites in BmAtg4. Single mutation of acetylation sites at K237 and K269, but not K125, partially decreased acetylation of BmAtg4; similar to the triple mutation, double mutation of acetylation sites at K237 and K269 totally abolished acetylation of BmAtg4 (Fig. 6G–G′). Double mutation of BmAtg4 acetylation sites at K237 and K269 led to its completely cytoplasmic localization and premature autophagy induction (Fig. 6H, I–I′). In addition, *BmHDAC1* overexpression led to the deacetylation of BmAtg4-c-Myc, showing the deacetylation of BmAtg8–PE ubiquitin-like system by BmHDAC1 (Fig. 6J). Overall, deacetylation of the components in BmAtg8–PE ubiquitin-like system at lysine residues leads to their nucleo-cytoplasmic translocation.

It is known that the components of LC3/Atg8–PE ubiquitin-like system, such as ATG7 and LC3 are sequestered in the nucleus by acetylation in mammals^11^^,^^12^^,^^19^, however, no information is available for ATG4. Results showed that *Homo sapiens ATG4b* (*HsATG4b*) overexpression notably promoted starvation-induced LC3-II formation; moreover, *HsATG4b* overexpression directly induced SQSTM1/p62 degradation and LC3-II formation (Supplementary Fig. S7A, B). Further analysis revealed that starvation induced the deacetylation and nucleo-cytoplasmic translocation of HsATG4b-HA in human embryonic kidney (HEK293) cells (Supplementary Fig. S7C, D). Four acetylation sites were identified in HsATG4b by MS analysis, of which only one site at K137 was conserved with BmAtg4 at K125 (Supplementary Figs. S6B, C and S7E). Mutation of single acetylation site partially decreased, while quadruple mutation of acetylation sites completely abolished, the acetylation of HsATG4b-HA, and consequently resulted in its completely cytoplasmic localization (Supplementary Fig. S7F, G). Moreover, quadruple mutation of HsATG4Bb-HA acetylation sites led to the degradation of SQSTM1/p62 and formation of LC3-II under nutrient-rich conditions, and further promoted starvation-induced autophagy (Supplementary Fig. S7H). Finally, *HsHDAC1* overexpression led to the deacetylation of HsATG4b (Fig. S7I). Taken together, our results show that human HsATG4b is deacetylated and shuttled from the nucleus to the cytoplasm to participate in autophagic process under starvation conditions.

**Fig. 7:**
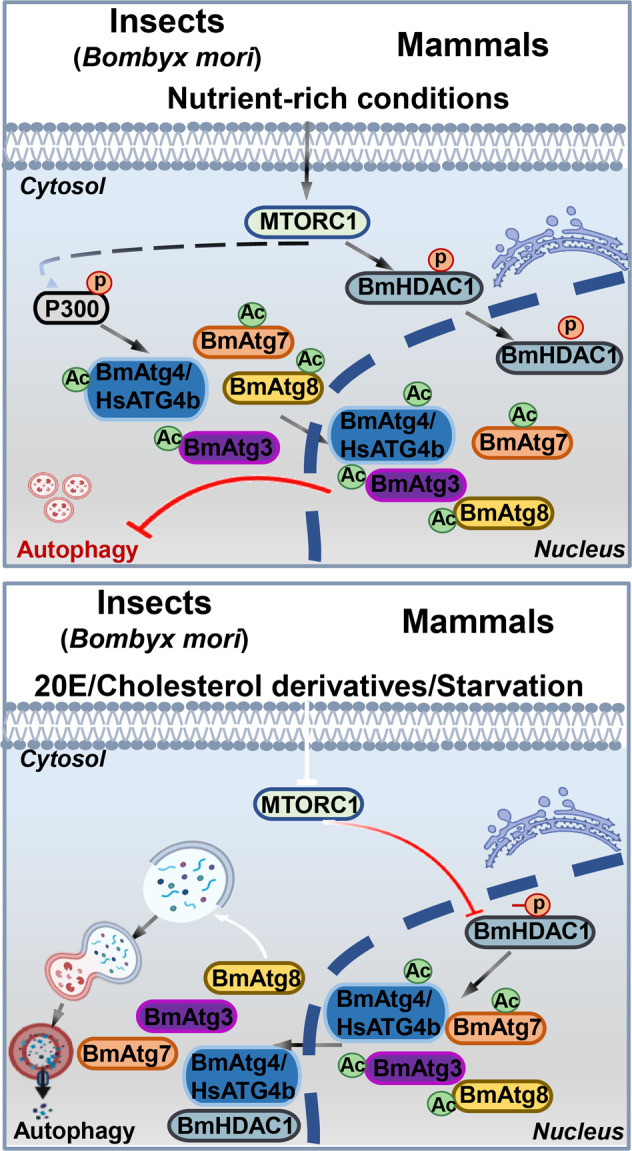
A model for the regulation of autophagy mediated by acetyltransferase/deacetylase in *B. mori* and mammals. Components of the LC3/Atg8–PE ubiquitin-like system are acetylated by acetyltransferase, such as BmP300, and then translocated from the cytoplasm to the nucleus under nutrient-rich conditions. Simultaneously, high MTOR activity phosphorylates the histone deacetylases BmHDAC1/HsHDAC1, resulting in their nuclear localization. 20E, cholesterol derivatives, and starvation, which inhibit MTOR signaling, leads to the dephosphorylation of BmHDAC1/HsHDAC1, and facilitates the deacetylation of Atg proteins from LC3/Atg8–PE ubiquitin-like system, subsequently promoting their nucleo-cytoplasmic translocation and autophagy occurrence in both *B. mori* and mammals.

Collectively, our data demonstrate that P300-mediated acetylation arrests the components of LC3/Atg8–PE ubiquitin-like system in the nucleus of both silkworm and humans under nutrient-rich conditions. After deacetylation by HDAC1 (which is activated after dephosphorylation induced by 20E/cholesterol derivatives and starvation), they are translocated from the nucleus to the cytoplasm, thus facilitating autophagy induction (Fig. 7).

## Discussion

### Regulation of autophagy by acetylation/deacetylation is evolutionarily conserved between insects and mammals

As introduced at the beginning, acetylation/deacetylation of the Atg8–PE ubiquitin-like system displays controversial function in autophagy regulation in yeast and mammals. In yeast, Atg3 and Atg8 are acetylated under nutrient-rich conditions. The acetylase ESA1 facilitates autophagy by catalyzing hyperacetylation of Atg3 under starvation when Atg8 is deacetylated; in contrast, the deacetylase Rpd3 inhibits autophagy^24^. In mammals, the deacetylases HDAC1 (homologous to yeast Rpd3), HDAC6, and SIRT1 facilitates autophagy, while the acetylase P300 inhibits autophagy^18^^,^^27^^,^^28^^,^^36^. Importantly, P300/SIRT1-mediated acetylation/deacetylation regulates LC3 subcellular localization, and thus autophagic activities of the Atg8–PE ubiquitin-like system^19^^,^^29^.

The regulatory mechanisms of Atg proteins by acetylation are poorly understood in insects. Although knockdown of *acetyl-coenzyme A synthase* in *Drosophila* brain has been shown to enhance autophagy and lifespan, the precise mechanism is not well documented^23^. Here, we demonstrate that the components of BmAtg8–PE ubiquitin-like system are necessary for BmAtg8–PE conjugation and autophagy induction in *B. mori*. BmP300 acetylates the components of the BmAtg8–PE ubiquitin-like system and sequesters them in the nucleus, thus inhibits autophagy in *B. mori*. Conversely, once deacetylated by BmHDAC1, these proteins are transferred from the nucleus to cytoplasm and promote autophagy. Similar to the mammalian LC3, here the BmAtg8 is studied in detail, including the acetylation sites; and so does BmAtg3. Notably, BmAtg4, the sole cysteine protease in this ubiquitin-like system, has been identified as an acetylated and nuclear localized protein under nutrient condition. Similar to BmAtg4, human ATG4b is subjected to deacetylation and nucleo-cytoplasmic translocation during autophagy induction. Mammalian ATG7 is acetylated by P300 under nutrient-rich conditions, but its subcellular localization and precise acetylation site are not observed. We did not study the details for too many acetylation sites of BmAtg7 were found. Taken together, results show a consistent acetylation pattern of the Atg8–PE ubiquitin-like system components in response to nutrient status in *B. mori*, displaying a mechanism which is evolutionarily conserved between insects and humans.

### Transcriptional and posttranslational regulation of autophagy in insects

In insects, autophagy is mainly regulated at both transcriptional and posttranslational levels. Nutrients and energy are universal upstream signals to elicit autophagy^1^. In insects, the exclusively present development regulator 20E regulates autophagy by inducing *Atg* gene expression after binding to the EcR-USP receptor^14^^,^^30^. In mammals, transcription factor forkhead box O3 (FoxO3), which is inhibited by insulin signaling, induces a set of *Atg* gene expressions^37^. In *Helicoverpa armigera*, 20E upregulates the transcriptional activity of *FoxO*, as well as its nuclear localization, thus autophagy promotion^38^. *dFOXO* mutation significantly reduced the transcription of *Atg1* and *Atg18* in *D. melanogaster*, indicating the involvement of FOXO in autophagy induction at transcriptional level in insects^39^.

In addition, 20E also modulates autophagy by direct antagonizing insulin/insulin-like growth factor signaling and activating the AMP-activated protein kinase–protein phosphatase 2A axis, which both lead to the inhibition of MTOR activity and the initiation of autophagosome formation through phosphorylation modification of Atg1/Atg13 protein complex^14^^,^^30^^,^^40^. Recently, we demonstrated that dephosphorylation of BmHDAC1 and its human homolog HsHDAC1 leads to their nucleo-cytoplasmic translocation after inhibition of MTOR signaling along with autophagy promotion^34^. Here, the precise mechanism of autophagy regulated by acetylation is revealed as that *B. mori* acetyltransferase BmP300 and deacetylase BmHDAC1 oppositely regulate the acetylation levels and subcellular localization of Atg proteins in BmAtg8–PE ubiquitin-like system, and thus autophagy upregulation. In general, the multiple signals including nutrient, energy, and 20E serve as upstream regulators of autophagy through induction of *Atg* gene expression and acetylation of ATG proteins in insects (Fig. S8).

Notably, the nuclear localization of BmAtg3 and BmAtg8 are accumulated after their mutual RNAi or knockout treatment, which are also similar to that of *BmAtg4* or *BmAtg7* RNAi. These results indicate that there may exist an inner operating mechanism of BmAtg8–PE ubiquitin-like system to maintain the autophagic flux or a feedback between the ubiquitin-like system and autophagy induction. Besides, whether and how other Atg proteins are regulated by acetylation, as well as the autophagic function of other acetylases and deacetylases in insects, deserve further investigation. Several PTMs in addition to phosphorylation and acetylation of Atg protein are reported to regulate their autophagic activities in yeast and mammals^18,22^, thus whether there are unrevealed PTMs on regulating autophagy is waiting for further exploration.

## Materials and methods

### Animals and cell culture

Silkworms (Dazao) were reared with fresh mulberry leaves at 25 °C under 14 h light/10 h dark cycle. BmN cells derived from *B. mori* ovary were cultured at 28 °C in Grace’s Insect Medium (Sigma-Aldrich, MO, USA, G9771) supplied with 10% heat-inactivated fetal bovine serum (AusGeneX, Molendinar, Australia, FBS500-S); HEK293 cells (ATCC, CRL-1573^TM^) were cultured in DMEM medium (Thermo Fisher, MA, USA, 10569044) supplemented with 5% fetal bovine serum. *Escherichia coli* DH5α (TIANGEN, Beijing, China, CB101-03) was used for subcloning, and *E. coli* BL21 (TIANGEN, Beijing, China, CB105-02) was used for prokaryotic expressions of proteins.

### Plasmid construction and transfection

Full-length of *BmAtg3*, *BmAtg4*, *BmAtg7*, and *BmAtg8* were cloned from total cDNA obtained from prepupal *B. mori* fat body and fused with c-Myc, His, V5, HA, or FLAG, respectively. Then they were inserted into the multiple cloning sites of pIEX4 vectors (Novagen, Beijing, China, 71235-2). The *HsATG4a*, *b*, *c*, *d* fused with tag sequence were cloned and constructed into pcMV3 vectors (Sino Biological, Beijing, China, HG22147-UT, HG20407-CY, HG15081-CM, and HG15537-CY). BmN or HEK293 cells were transiently transfected with the respective plasmids using FuGENE® HD Transfection Reagent (Promega, WI, USA, E2311) or TransIntro^TM^ EL Transfection Reagent (TransGen Biotech, Beijing, China, FT201-01), according to the manufacturer’s instruction.

### Production of recombinant proteins

Full-length *BmAtg3*, *BmAtg8*, *BmAtg4*, and *BmAtg7* were constructed into the ppSUMO vector fused with His and SUMO tags, respectively. BL21 (DE3) cells were transformed with these plasmids and then cultured in 600 mL Luria-Bertani medium at 37 °C until OD_600_ reached 0.5–0.6. Subsequently, protein expression was induced by incubating the cells with 0.5 mM isopropyl-β-d-1-thiogalactopyranoside at 25 °C for 10 h. Then, the cells were harvested by centrifugation at 5000 × *g* for 5 min. The pellet was resuspended in phosphate-buffered saline (PBS; NaCl 8 g/L, KCl 0.2 g/L, Na_2_HPO_4_ 1.42 g/L, and KH_2_PO_4_ 0.27 g/L, pH 7.0) supplemented with 1 mg/mL lysozyme and 1 mM PMSF (phenyl methane sulfonyl fluoride). The cell suspension was incubated for 30 min on ice and then lysed by sonication (Vibracell VCX 750 Sonifier, Sonics Inc, Japan). The supernatant was collected by centrifugation at 12,000 × *g* for 30 min, and then was passed through a Nickel-charged Sepharose column (60 mL, GE Healthcare, Illinois, USA) pre-equilibrated with the binding buffer. After washing with binding buffer (20-fold column volume), the resin column was eluted using 10 mL imidazole elution buffer at different concentrations.

### LC–MS/MS

The acetylation sites in BmAtg3, BmAtg4, BmAtg7, BmAtg8, and HsATG4b were identified by liquid chromatography associated with MS. Recombinant proteins were subjected to trypsin-mediated digestion. The digested peptides were dissolved in 0.1% formic acid and 2% acetonitrile, directly loaded onto a reversed-phase analytical column (Thermo Fisher, MA, USA, 75 μm i.d. × 150 mm, packed with Acclaim PepMap RSLC C18; 2 μm, 100 Å, nanoViper), and separated by a gradient elution buffer. The peptides were analyzed by Nano-Spray-Ionization (NSI) linear ion trap tandem MS (Thermo Fisher, MA, USA, Scientific Q Exactive^TM^). Protein identification was performed with MASCOT software (Mascot Soft Web Solution Pvt. Ltd., Uttarakhand, India) and using Uniprot (https://www.uniprot.org/) search for *B. mori*.

### Chemicals and starvation treatments

Twelve hours before initiation of wandering stage (IW), larvae were injected with different doses of TSA (MCE, NJ, USA, HY-15144; 0, 3, 6, 12, and 18 μg/larva) and CTB (Sigma-Aldrich, MO, USA, C6499; 0, 5.5, 11, 22, and 33 μg/larva). Larvae at 5L3D were injected with different doses of C646 (MCE, NJ, USA, 328968-36-1; 0, 4.5, 9, 18, 27, and 36 μg/larva) or 10 μg/larva 20E. The fat body was collected 24 h later for further analyses. BmN cells were treated with different concentrations of 20E (Sigma-Aldrich, MO, USA, H5142; 1, 2.5, and 5 μM) for 6 h, or starved for 2, 4, or 8 h. *BmAtg3*, *BmAtg4*, *BmAtg7*, *BmAtg8*, or *HsATG4b* overexpressed cells were treated with TSA (20 μM) for 2 h, C646 (800 nM) for 6 h, or CTB (20 μM) for 2 h, and then collected for further analyses.

### RNAi knockdown

For RNA interference, the templates of *BmAtg4* and *BmAtg7* were amplified by PCR from total cDNA obtained from *B. mori* fat body. Double-stranded RNA (dsRNA) was generated using the T7 RiboMAX^TM^ Express RNAi system (Promega, WI, USA, P1700), according to the manufacturer’s instructions. dsRNA (50 μg/larva) was injected into the larvae 12 h before IW, with *egfp* dsRNA injection as control. The fat body was collected 24 h after injection for further analyses. All primers used in this study are listed in Table S1.

### Gene editing in silkworms

*Cas9* mRNA and *BmAtg3* or *BmAtg8* sgRNA were synthesized, using mMESSAGE mMACHINE T7 Kit (Thermo Fisher, MA, USA, AM1344) and MEGAscript™ T7 Kit (Thermo Fisher, MA, USA, AM1334), respectively. A mixture of *Cas9* mRNA (300 ng/μL) and *BmAtg3* sgRNA (150 ng/μL) was injected into silkworm eggs within 6 h after oviposition using a microinjector (Eppendorf, InjectMan®4, Hamburg, Germany). *Cas9* mRNA injection was used as control. The embryos were incubated at 25 °C in a humidified incubator until hatching, and then raised with fresh mulberry leaves at 25 °C under 14 h light/10 h dark cycle. The fat body was collected 24 h after IW for further analyses. The genomic segments with length near 500 bp for *BmAtg3* and *BmAtg8* containing the knockout site were cloned and sequenced.

### Immunoprecipitation and western blotting

BmN cells overexpressing *BmAtg3*, *BmAtg4*, *BmAtg7* or *BmAtg8*, and HEK293 cells overexpressing *HsATG4a*, *b*, *c* or *d* were lysed in NP-40 lysis buffer (50 mM Tris-HCl, pH 7.4, 1% NP-40, 150 mM NaCl, 2 mM EDTA, 1 mM dithiothreitol, and 10% glycerol) supplemented with a complete protease inhibitor cocktail (Roche, Basel, Switzerland, 0469313201). The supernatant was incubated with tag antibodies at 4 °C for 4 h, and then incubated with protein A/G agarose beads (Thermo Fisher, MA, USA, 20421) overnight according to a standard immunoprecipitation procedure^41^.

Western blotting was performed using antibodies against BmAtg3 (Abclonal Technology, Wuhan, China, 017751D; 1:3000), BmAtg8 (1:3000), BmSqstm1 (Abclonal Technology, Wuhan, China, A18679; 1:3000)^30^^,^^35^, c-Myc (Sigma-Aldrich, MO, USA, 11667149001; 1:1000), His (TransGen Biotech, Beijing, China, HT-501; 1:1000), FLAG (Invitrogen, CA, USA, MA1-142; 1:1000), V5 (TransGen Biotech, Beijing, China, HT-401; 1:1000), HA (Santa Cruz Biotechnology, Texas, USA, sc-7392; 1:2000), SQSTM1/p62 (Cell Signaling Technology, MA, USA, 5114; 1:1000), LC3B (Abcam, Cambridge, UK, 192890; 1:2000), and acetylated-lysine (Cell Signaling Technology, MA, USA, 9441; 1:2000). LMNB/Lamin B1 (Bioworld Technology, Illinois, USA, AP6001; 1:5000), TUBA1A/tubulin alpha 1a (Beyotime Biotechnology Co., Ltd., Shanghai, China, AF0001; 1:5000), and ACTB/actin beta (ProMab Biotechnology, Hunan, China, 20270; 1:1000) were used as reference proteins^30^^,^^34^^,^^41^. All western blotting images were taken with a Tanon-5200 Chemiluminescent Images System. ImageJ software (National Institutes of Health, Image processing and analysis in java) was used to perform quantitative measurements of western blots from three independent biological repeats.

### Immunofluorescent staining

The fat body was collected and fixed in 4% paraformaldehyde in 0.1 M PBS (pH 7.5) at 4 °C for 6 h, and further fixed with 4% paraformaldehyde (PBS, pH 10.4) at 4 °C overnight. The samples were subjected to a standard procedure for immunofluorescent staining^42^: an overnight incubation at 4 °C with a primary antibody against BmAtg3 (1:100) or BmAtg8 (1:100) preceded incubation for 2 h with a fluorescent secondary antibody conjugated with Alexa Flour 488 (Abcam, Cambridge, UK, ab150077; 1:200). Staining with DAPI (Beyotime Biotechnology Co., Ltd., Shanghai, China, C1005) for 15 min at 25 °C allowed the visualization of nuclei^42^.

Sterilized coverslips were placed into six-well plates (Guangzhou Jet Bio-Filtration Co., Ltd., TCP-010-006) during BmN or HEK293 cell plating. After preincubation for 24 h, cells were transiently transfected with the aforementioned *BmAtgs* or *HsATG4* genes, respectively, for 48 h, followed by different treatments, including chemicals and starvation. Cells were fixed in 4% paraformaldehyde (PBS, pH 10.4) at 4 °C for 30 min and then subjected to immunofluorescent staining with tag antibodies. Images were acquired using confocal fluorescence microscope equipped with an Olympus digital camera (FV3000, Olympus, Tokyo, Japan). To quantify Alexa Fluor 488-labeled BmAtg3 and BmAtg8, 150–200 fat body cells or a total of 30 overexpressed BmN cells from three independent biological repeats were recorded and analyzed, using ImageJ software^34^.

### Isolation of nuclear and cytoplasmic proteins

Fat body from day 2 of fifth larval instar (5L2D) and day 2 of prepupa (PP2) were then harvested for nuclear and cytoplasmic extraction using a NE-PERTM Nuclear Cytoplasmic Extraction Reagent Kit (Pierce, Texas, USA, 78833), according to the manufacturer’s instruction. The separated proteins were evaluated with western blotting.

### LysoTracker red staining

The fat body was collected, thoroughly washed with PBS (pH 7.0, 0.1 M), and then stained with LysoTracker Red dye (Invitrogen, CA, USA, L7528, 50 nM) for 5 min at 37 °C, as previously described^34^. All images were taken using confocal fluorescence microscope (Olympus FV3000). Three independent experiments were performed.

### Transmission electron microscopy analysis

The fat body was collected and fixed in 2.5% glutaraldehyde at least for 24 h at 4 °C, and then samples were processed, as previously described^30^. Specimens were analyzed under a TEM to observe autolysosomes and autophagosomes, three independent biological repeats were conducted.

### Quantitative real-time PCR (qRT-PCR)

Total RNA was extracted using RNAiso Plus reagent (TaKaRa, Dalian, China, 9108). cDNA was obtained by PrimeScript^TM^ RT reagent Kit (TaKaRa, Dalian, China, RR047A). qRT-PCR was performed as previously described^30,42^. *Rp49* was used as reference gene. PCR primers used are listed in Table S1.

### Statistics analysis

One-way analysis of variance was performed by SPSS, *p < 0.05, **p < 0.01, and ***p < 0.001. The values are shown as mean ± standard deviation of three independent experiments. Ten animals were used for each repeat, and three biological replicates were conducted.

## Supplementary information

Supplemental Figure 1

Supplemental Figure 2

Supplemental Figure 3

Supplemental Figure 4

Supplemental Figure 5

Supplemental Figure 6

Supplemental Figure 7

Supplemental Figure 8

Table S1

Supplementary Figure Legends
