# Supplementary figures and images for: P300/HDAC1 regulates the acetylation/deacetylation and autophagic activities of LC3/Atg8–PE ubiquitin-like system
Source: Cell Death Discov. 2021 May 31;7:128. doi: 10.1038/s41420-021-00513-0 (PMC8166822; doi:10.1038/s41420-021-00513-0)

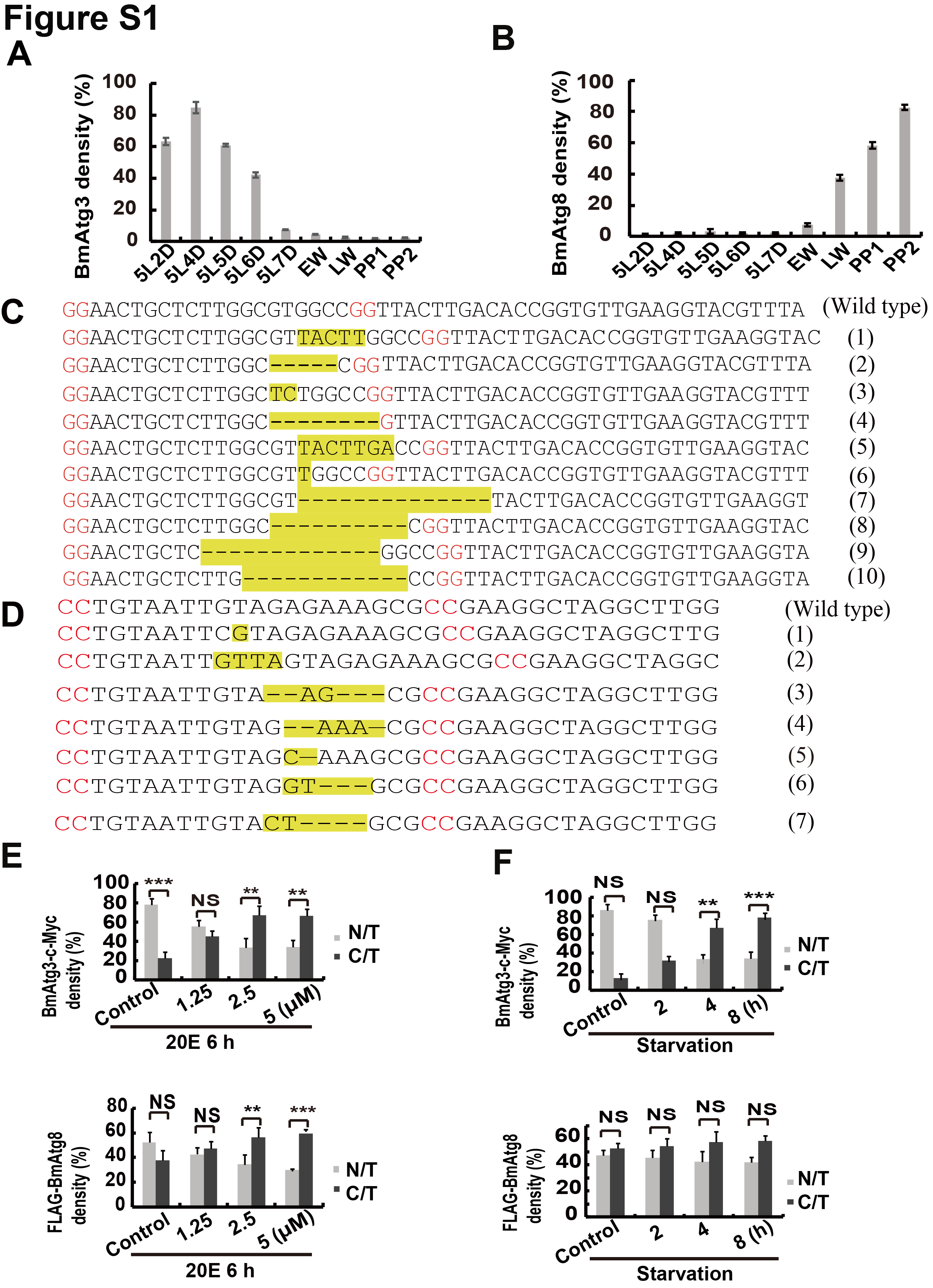

Supplement: Supplementary file 1 — Supplemental Figure 1 [file 41420_2021_513_MOESM1_ESM.png]

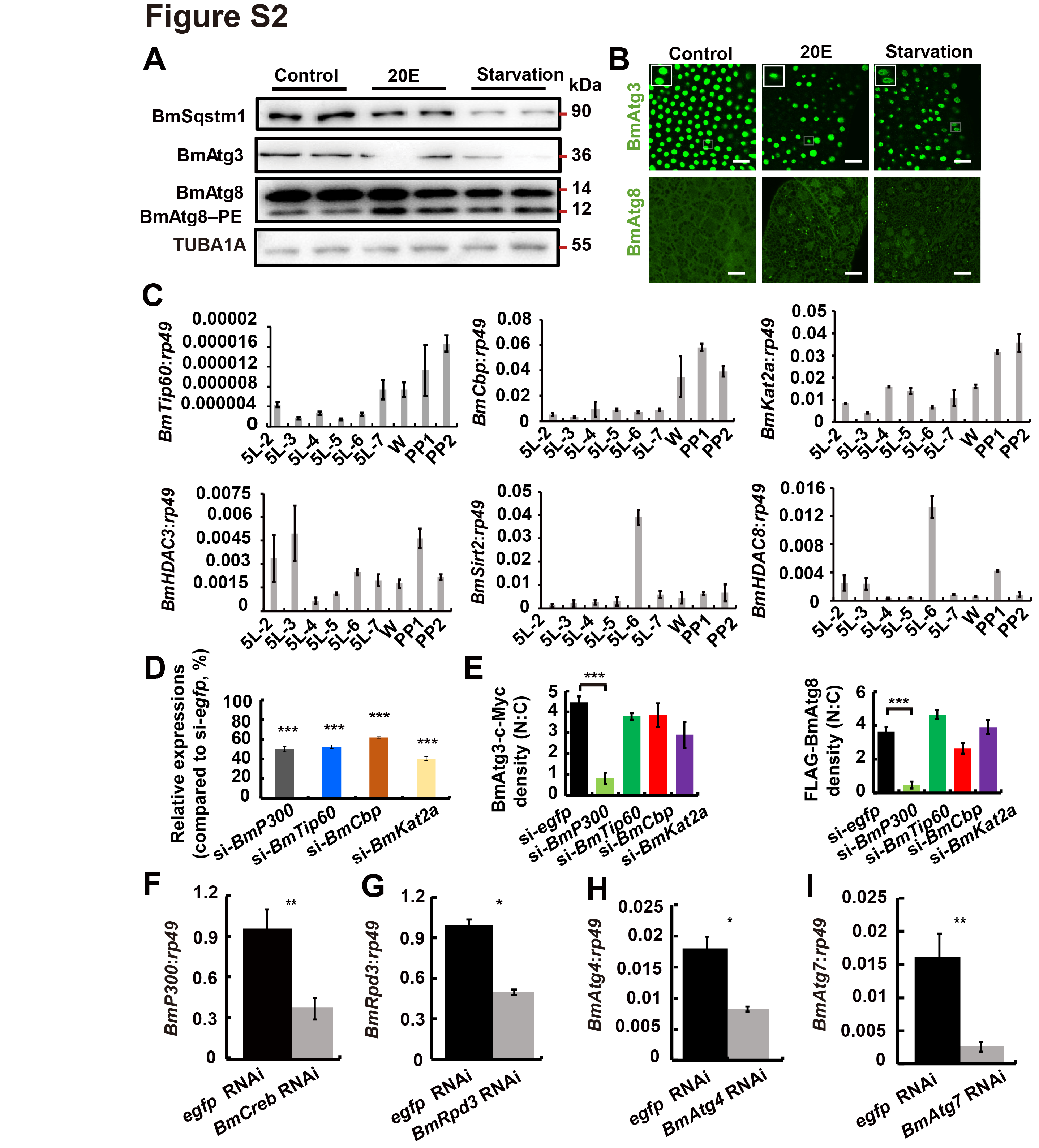

Supplement: Supplementary file 2 — Supplemental Figure 2 [file 41420_2021_513_MOESM2_ESM.png]

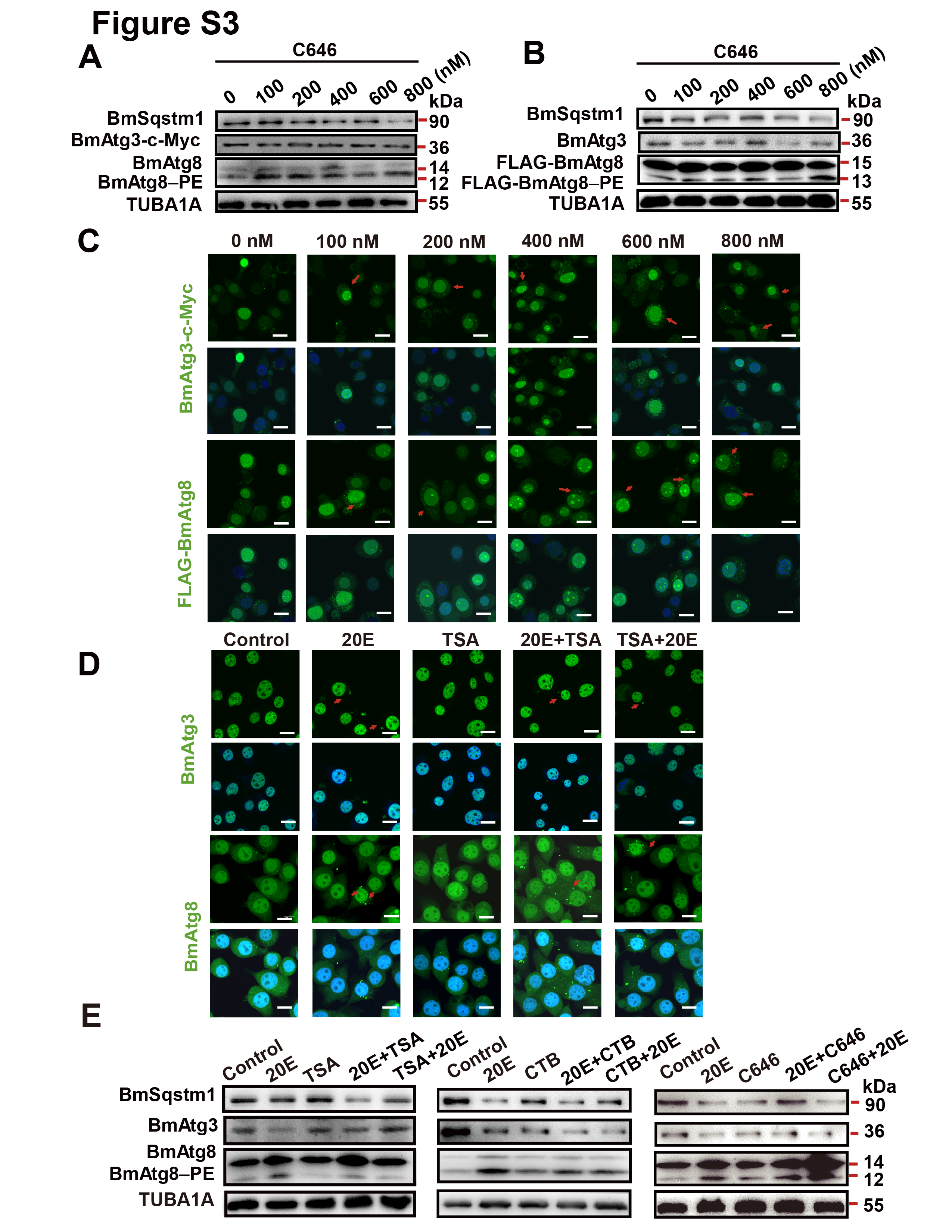

Supplement: Supplementary file 3 — Supplemental Figure 3 [file 41420_2021_513_MOESM3_ESM.png]

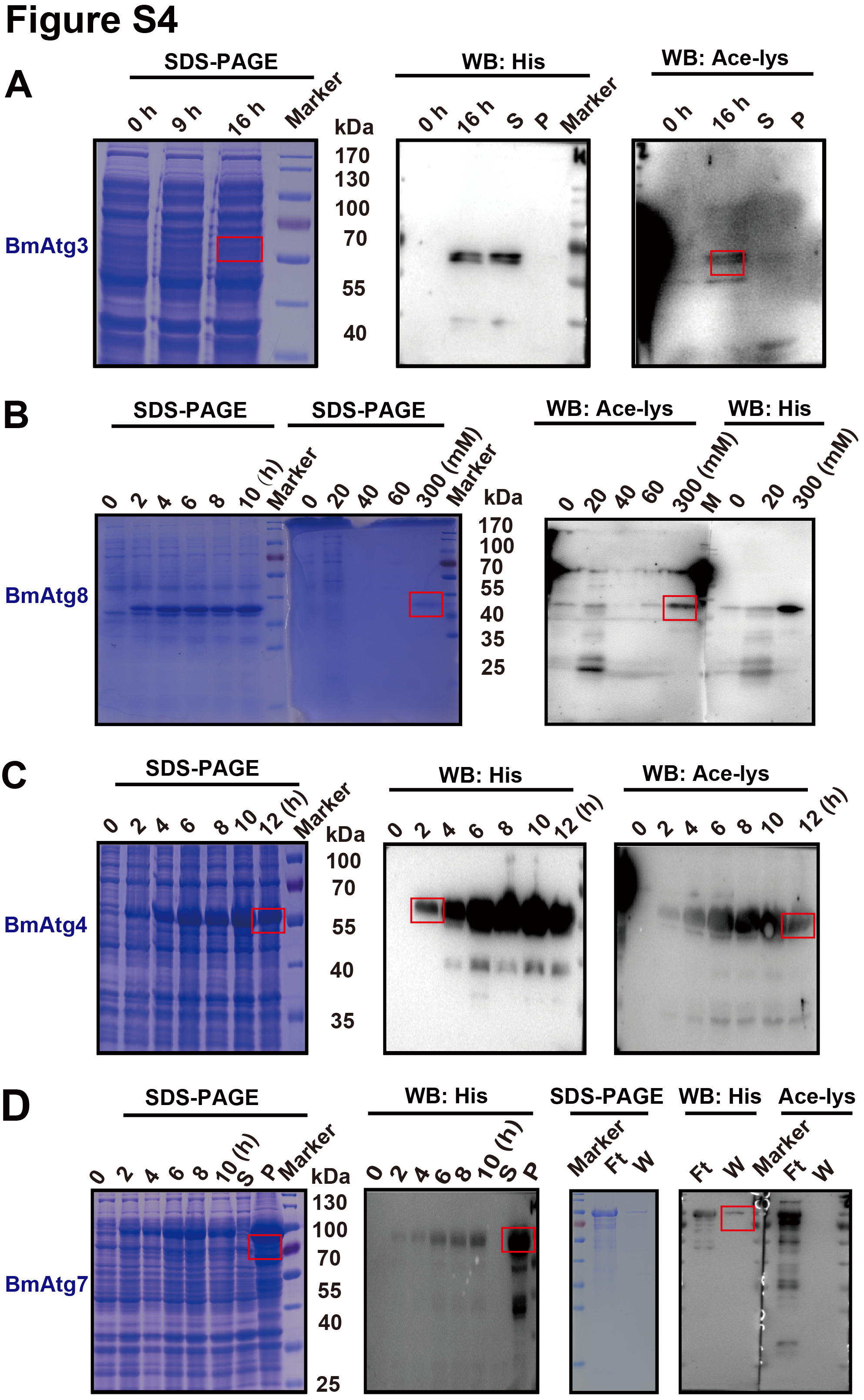

Supplement: Supplementary file 4 — Supplemental Figure 4 [file 41420_2021_513_MOESM4_ESM.png]

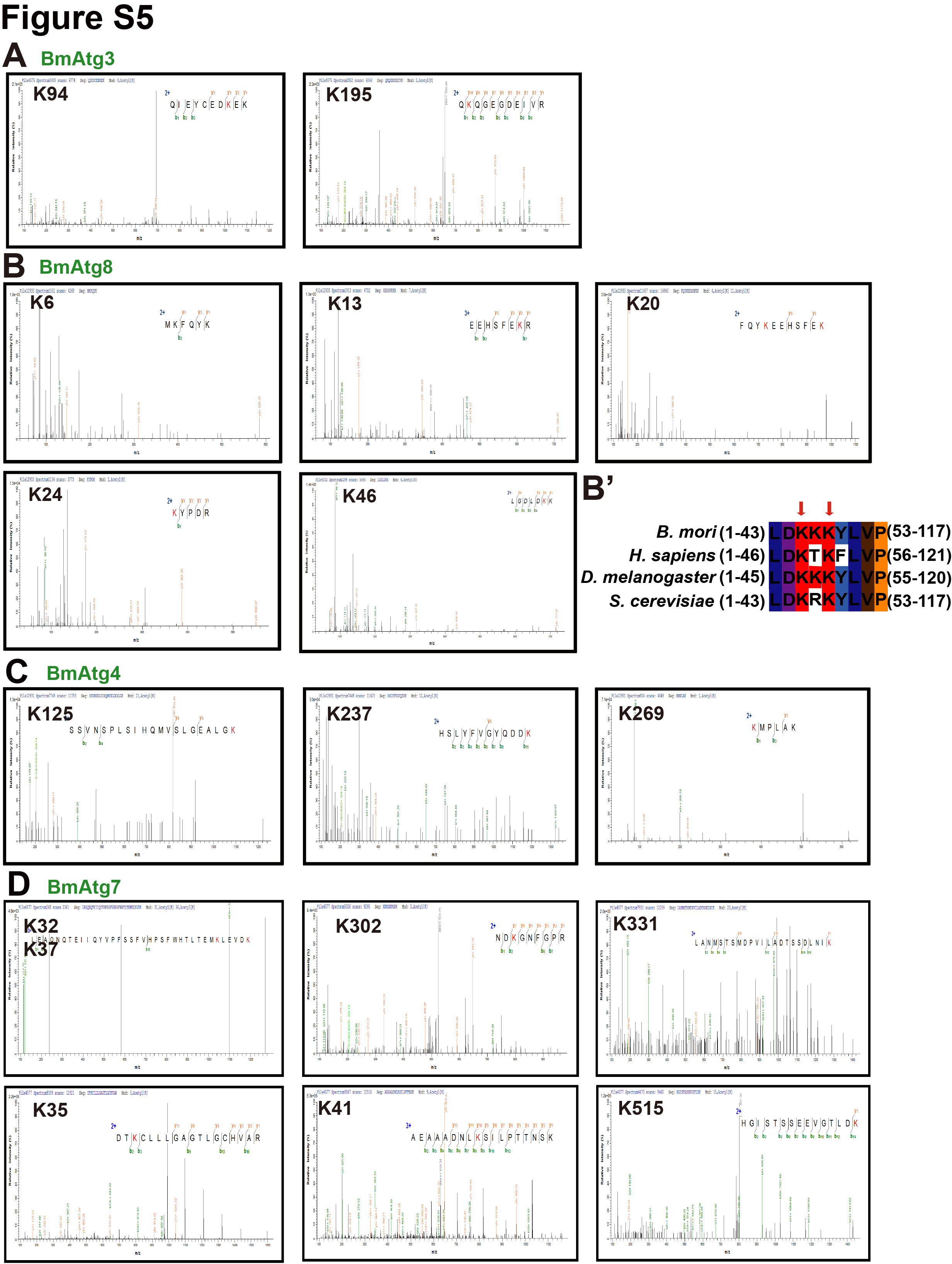

Supplement: Supplementary file 5 — Supplemental Figure 5 [file 41420_2021_513_MOESM5_ESM.png]

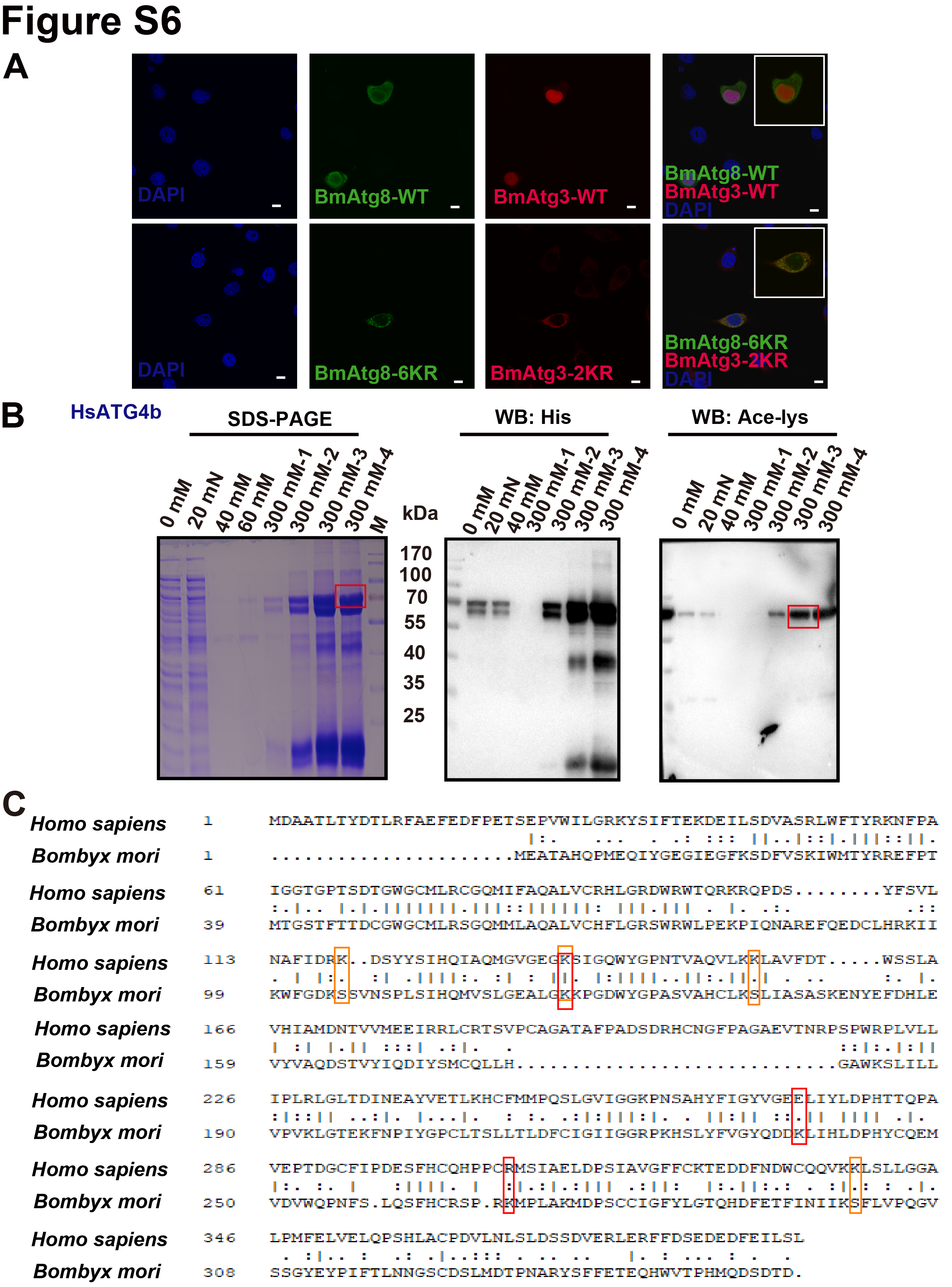

Supplement: Supplementary file 6 — Supplemental Figure 6 [file 41420_2021_513_MOESM6_ESM.png]

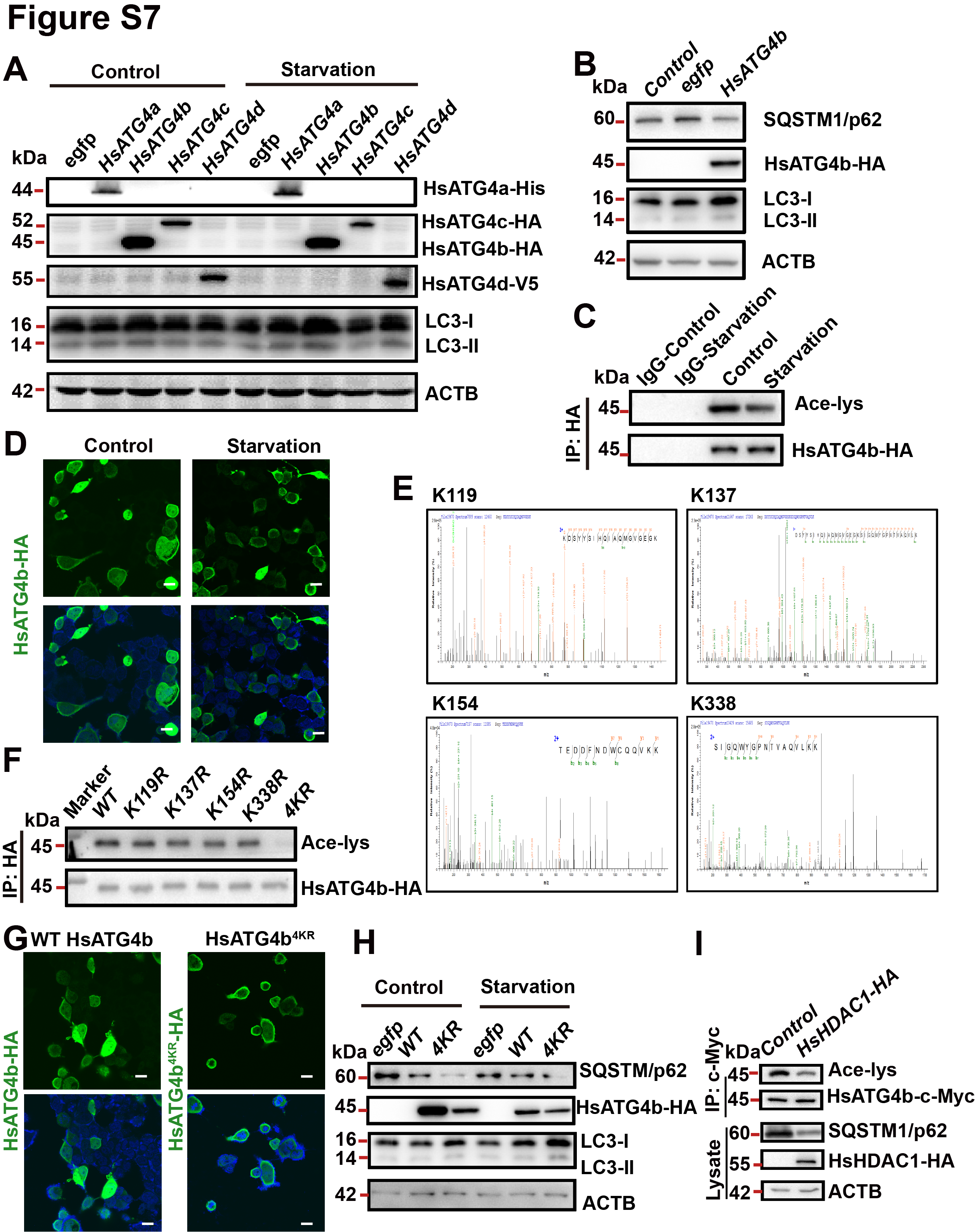

Supplement: Supplementary file 7 — Supplemental Figure 7 [file 41420_2021_513_MOESM7_ESM.png]

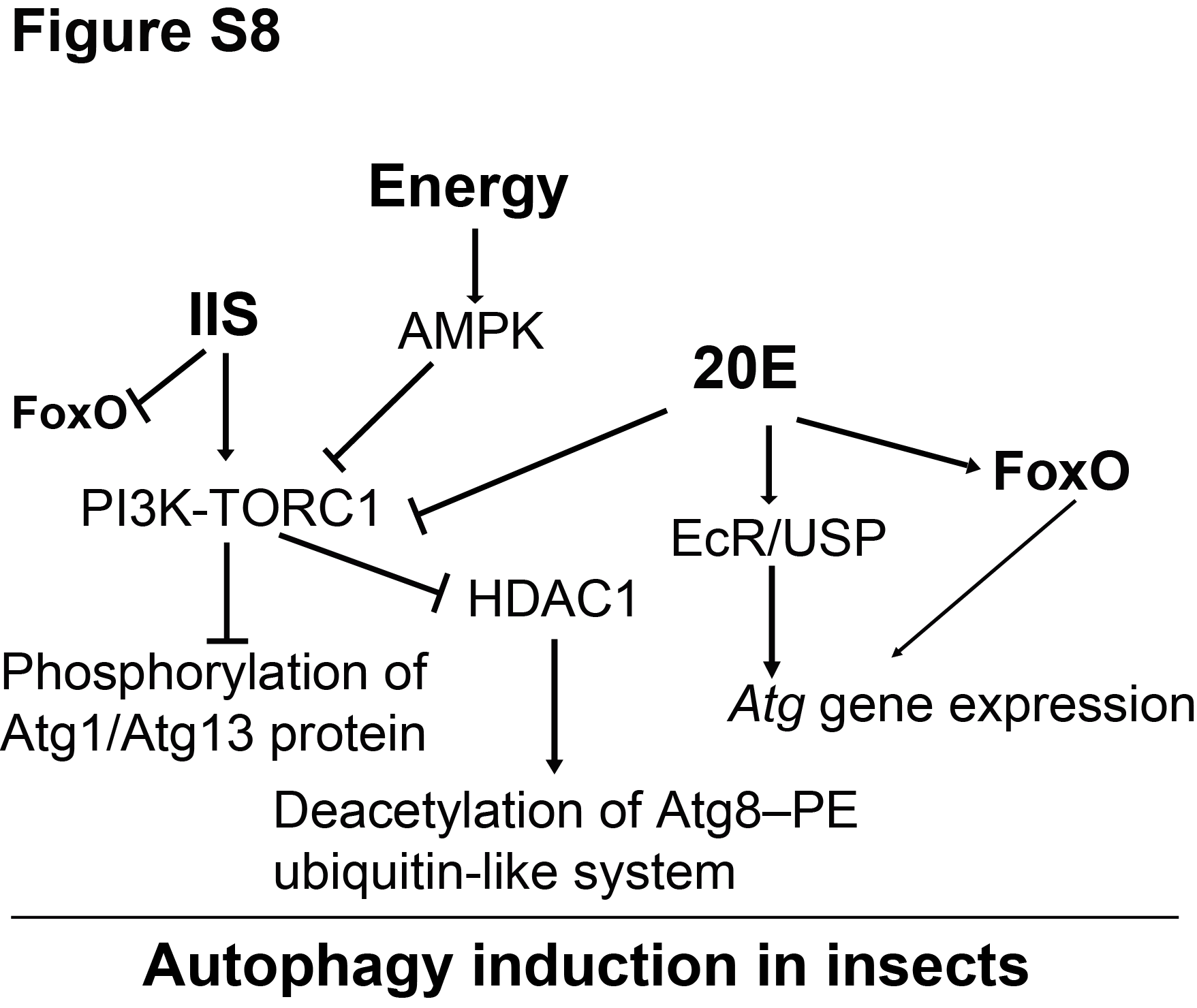

Supplement: Supplementary file 8 — Supplemental Figure 8 [file 41420_2021_513_MOESM8_ESM.png]
