# Supplementary material for: P300/HDAC1 regulates the acetylation/deacetylation and autophagic activities of LC3/Atg8–PE ubiquitin-like system
Source: Cell Death Discov. 2021 May 31;7:128. doi: 10.1038/s41420-021-00513-0 (PMC8166822; doi:10.1038/s41420-021-00513-0)
Supplement: Supplementary file 9 — Table S1 [file 41420_2021_513_MOESM9_ESM.docx]

**Table S1 Primers and siRNA sequence**

| **Gene name Genbank number Primer sequence**  pIEX4-Atg3-F NP_001135961.1 CATGCCATGGCAATGCAAAGTGTAATAAACACAGTAA  pIEX4-Atg3-R CGAGCTCTTAGTTAATCGAGAAATTCTGTGTG  pIEX4-Atg3-Myc-His-R CGAGCTCTTAATGGTGATGGTGATGATGAGATGCCAGATCTT  CTTCAGAAATAAGTTTTTGTTCGTTAATCGAGAAATT  pIEX4-Atg8-F NM_001046779.1 ATGAAATTCCAATACAAAGAAG  pIEX4-Atg8-FLAG-F GGAAGATCTGACTACAAGGACGACGATGACAAGATGAAATT  CCAATACAAAG  pIEX4-Atg8-R CGGGGTACCTTAATTTCCATAGACATTTTCGTC  pIEX4-Atg4-F XM_021346895 CATGCCATGGCAATGGAAGCAACTGCACATCAACCAATGGAAC  pIEX4-Atg4-R TTGGCGCGCCTAAGTCTGTATCGCTGTCTTGCATATGTG  pIEX4-Atg4-V5-R TTGGCGCGCCTAACGTAGAATCGAGACCGAGGAGAGGGTTAG GGATAGGCTTACCGTCTGTATCGCTGTCTTGCATATGTG  pIEX4-Atg4-Myc-F CATGCCATGGCAATGGAACAAAAACTTATTTCTGAAGAAGATC  TGGAAGCAACTGCACA  pIEX4-Atg7-F KY608887.1 CATGCCATGGCAATGTTGGAAGCACAAAACCAAACAGAA  pIEX4-Atg7-R TTGGCGCGCCCTAATCTTCTTCATCGGTTAATGTCTT  pIEX4-Atg7-HA-R TTGGCGCGCCCTAAGCGTAATCTGGAACATCGTATGGGTAATCT TCTTCATCGGTTAATGTCTT  pIEX4-HDAC1-F XP_004931440.2 CATGCCATGGCAATGTCTATGCAACCGCACAGTAAGAAAAG  pIEX4- HDAC1-R TCAGGGATTCGGTGGCATGTCCTTCTTT  pIEX4-HDAC1-HA-R CGGGGTACCTCAAGCGTAATCTGGAACATCGTATGGGTAGGGA TTCGGTGGCATGTCCTTCTTT  [pCMV3-ATG4b-F](https://cn.sinobiological.com/category/pcmv3-c-his-vector) NM_013325.4 GGGGTACCATGGACGCAGCTACTCTGACCT  [pCMV3-ATG4b-Myc-R](https://cn.sinobiological.com/category/pcmv3-c-his-vector) TTGCGGCCGCTCACAGATCTTCTTCAGAAATAAGTTTTTGTTCAA  GGGACAGGATTTCA  Atg3-K94R-F TGAAGATCGAGAGAAAGTGATAGAAGATGAAAACG  Atg3-K94R-R CGTTTTCATCTTCTATCACTTTCTCTCGATCTTC  Atg3-K195R-F GATAAGAAACAGCGCCAAGGAGAAGGTGATGAG  Atg3-K195R-R CTCATCACCTTCTCCTTGGCGCTGTTTCTTATC  Atg8-K6-F ATGAAATTCCAATACCGAGAAG  Atg8-K6-R ATGTTCTTCTCGGTATTGGAATTT  Atg8-K13-F CCCTCAGCCTTTCTGCGCTCAAAT  Atg8-K13-R CTCAGCCTTTCTGCGCTCAAATG  Atg8-K20-F GGGCGAACGAATCCGCAGGAAATAT  Atg8-K20-R CTGGATATTTCCTGCGGATTCGTT  Atg8-K24-F AAAATCCGCAGGCGATATCCAG  Atg8-K24-R GCGATCTGGATATCGCCTGCGGATT  Atg8-K46K48-F ACCTCGACCGAAAGCGATATTTAGT  Atg8-K46K48-R TAAATATCGCTTTCGGTCGAGGTCT  Atg4-K125-F TTTAGGCCGCAAGCCAGGTGAT  Atg4-K125-R CCAATCACCTGGCTTGCGGCCTAAA  Atg4-K237-F ATCAAGATGACCGACTCATTCA  Atg4-K237-R GATGAATGAGTCGGTCATCTTGAT  Atg4-K269-F TTCTCCAAGACGAATGCCACTT  Atg4-K269-R GCAAGTGGCATTCGTCTTGGAGA  ATG4b-K357-F CAGGCGAGACAGTTACTACTCCATT  ATG4b-K357-R ACTGTCTCGCCTGTCGATGAATGC  ATG4b-K411-F AAGGCCGATCCATAGGCCAGTGGTA  ATG4b-K411-R TGGATCGGCCTTCGCCAACTCCCAT  ATG4b-K462-F TGAAGCGACTTGCTGTCTTCGATAC  ATG4b-K462-R AGCAAGTCGCTTCAGGACCTGGGCG  ATG4b-K1104-F TCAAACGACTGTCTCTGCTTGGAGG  ATG4b-K1104-R GACAGTCGTTTGACTTGCTGGCACC  ppSUMO-Atg3-F CCGGAATTCATGCAAAGTGTAATAAACACA  ppSUMO-Atg3-R CCCAAGCTTTTACAGATCTTCTTCAGAAATAA  ppSUMO-Atg8-F CCGGAATTCATGTCGTTAACACGTCAAG  ppSUMO-Atg8-R CCCAAGCTTTTAATTTCCATAGACA  ppSUMO-Atg4-F CGAGCTCATGGAAGCAACTGCACATCAACC  ppSUMO-Atg4-R TAAAGCGGCCGCTTAGTCTGTATCGCTGTC  ppSUMO-Atg7-F CCGGAATTCATGTTGGAAGCACAAAACCAAACAGAA  ppSUMO-Atg7-R CCCAAGCTTCTAATCTTCTTCATCGGTTAATGTCTT  ppSUMO-ATG4b-F CCGGAATTCATGGACGCAGCTACTC  ppSUMO-ATG4b-R CCCTCGAGTCAAAGGGACAGGATTTCAAAGTC  Atg4-RNAi-F GGATCCTAATACGACTCACTATAGGAAGCACCTCATACCCCAC  Atg4-RNAi-R GGATCCTAATACGACTCACTATAGGTCTCCCAAACTCACCATCT  Atg7-RNAi-F GGATCCTAATACGACTCACTATAGGGAAGTCAATCCTCCCGACTA  Atg7-RNAi-R GGATCCTAATACGACTCACTATAGGGCAACAACGCCACCAGTATC  EGFP-RNAi-F GGATCCTAATACGACTCACTATAGGGAGAATGGTGAGCAAGGGC  EGFP-RNAi-R GGATCCTAATACGACTCACTATAGGGAGACTTGTACAGCTCGT  BmHDAC1-RNAi-F GGATCCTAATACGACTCACTATAGGATATTGACATAGATGTGCAT  BmHDAC1-RNAi-R GGATCCTAATACGACTCACTATAGGCGAGATACATTACGTATGGT  BmP300-RNAi-F XP_021204790.1 GGATCCTAATACGACTCACTATAGGTGGCCACAACAATCCTTTAG  BmP300-RNAi-R GGATCCTAATACGACTCACTATAGGTCCTGTTGCAGACTGCCCGT  rp49-S CAGGCGGTTCAAGGGTCAATAC  rp49-A TGCTGGGCTCTTTCCACGA  Atg4-S TACCTCAGGGTGTATCATCA  Atg4-A TAAGTCTGTATCGCTGTCTTG  Atg7-S GAGGCGAGATGGCTGC  Atg7-A CGAGGTGCTAATTCCGTG  Cbp-S XP_021206531.1 GTCTCATCCTCACATACG  Cbp-A CTCGGTCATCAGATTCAC  Kat2a-S XP_004922629.1 TGTAGCAGAAGTGAATAGG  Kat2a-A TTAGTGTTGTGGTCATCTT  Tip60-S XP_004928298.1 CTTCGTCACTGGTCAATGG  Tip60-A TCCGCTTCACCTTCTTGT  Sirt2-S NP_001036937.1 TGCCCTGGTGTTGTGAAG  Sirt2-A CATTGCTTGAAGTCCTCCTCTA  HDAC1-S AGTGTGGTGCTGATTCTCT  HDAC1-A AACAGATGTCTCGTAAGTCCAA  HDAC3-S XP_012552478.1 ACCAGTGATCTCCTATGTGAT  HDAC3-A GAAGCATCCAAGCCTATCG  HDAC8-S XP_021205467.1 GCATCATGGCAATGGTGTTCAAGA  HDAC8-A CCGTCACCGCAACCAATGT  P300-S GCAACAACAGCAACAACAACT  P300-A GATACTGGATTGGCGGTCAC  Atg8 sgRNA-F ATGAAATTCCAATACAAAGA  Atg8 sgRNA-R TTAATTTCCATAGACATTTTCG  Atg3 sgRNA-F ATGCAAAGTGTAATAAACACAGTA  Atg3 sgRNA-R AGTCAGTACACCAGTTTCTCG  *BmAtg3* sgRNA-S GGAACTGCTCTTGGCGTGGC  *BmAtg3* sgRNA-A GCCACGCCAAGAGCAGTTCC  *BmAtg8* sgRNA-S CCTGTAATTGTAGAGAAAGCG  *BmAtg8* sgRNA-A CGCTTTCTCTACAATTACAGG  *BmP300* siRNA-S ACGUUUGUGACGUUCAUGCCG  *BmP300* siRNA-A GCAUGAACGUCACAAACGUUG  *BmCbp* siRNA-S CAAUAUUAUUGUUGUUCUACG  *BmCbp* siRNA-A UAGAACAACAAUAAUAUUGUA  *BmKat2a* siRNA-S CGGUGCUGUUGUAGACGUAGA  *BmKat2a* siRNA-A UACGUCUACAACAGCACCGAG  *BmTip60* siRNA-S GGUGGUACAACUACUCCAAAG  *BmTip60* siRNA-A UUGGAGUAGUUGUACCACCUG |
| --- |
