# Supplementary material for: P300/HDAC1 regulates the acetylation/deacetylation and autophagic activities of LC3/Atg8–PE ubiquitin-like system
Source: Cell Death Discov. 2021 May 31;7:128. doi: 10.1038/s41420-021-00513-0 (PMC8166822; doi:10.1038/s41420-021-00513-0)
Supplement: Supplementary file 10 — Supplementary Figure Legends [file 41420_2021_513_MOESM10_ESM.docx]

**Fig. S1 A** Quantification of fluorescent BmAtg3 in Fig. 1A. **B** Quantification of fluorescent BmAtg8 in Fig. 1B. **C** The variation of *BmAtg3* from the genome after *BmAtg3* knockout. **D** The variation of *BmAtg8* from the genome after *BmAtg8* knockout. **E** Quantification of fluorescent BmAtg3 and BmAtg8 in the nucleus and the cytoplasm in Fig. 2C. **F** Quantification of fluorescent BmAtg3 and BmAtg8 in the nucleus and the cytoplasm in Fig. 2D.

**Fig. S2** Detections of autophagy, fluorescent staining of BmAtg3 and BmAtg8, gene mRNA levels, and quantification of fluorescent BmAtg3 and BmAtg8. **A** Protein levels of BmSqstm1, BmAtg3, and BmAtg8 after 20E treatment or starvation for 24 h in *B. mori* fat body. **B** Immunofluorescent staining of BmAtg3 and BmAtg8 after 20E treatment or starvation for 24 h. Scale bar: 10 micron. **C** mRNA levels of acetyltransferases *BmTip60*, *BmCbp*, and *BmKat2a,* and deacetylases *BmSirt2*, *BmHDAC3*, and *BmHDAC8* in *B. mori* fat body from 5L2D to PP2 stages. **D** mRNA levels of *BmP300*, *BmTip60*, *BmCBP*, and *BmKat2a* after their respective siRNA treatments for 24 h in BmN cells in Fig. 3A. **E** Quantification of fluorescent BmAtg3 and BmAtg8 in Fig. 3A’. **F** mRNA levels of *BmP300* after its RNAi treatment for 24 h in Fig. 3C. **G** mRNA levels of *BmHDAC1* after its RNAi treatment for 24 h in Fig. 3D. **H** mRNA levels of *BmAtg4* after its RNAi treatment for 24 h in Fig. 5A. **I** mRNA levels of *BmAtg7* after its RNAi treatment for 24 h in Fig. 5B.

**Fig. S3** Regulation of BmAtg3 and BmAtg8 by the inhibitors C646 and TSA in BmN cells. **A** Protein levels of BmSqstm1, endogenous BmAtg8, and BmAtg3-c-Myc after 100, 200, 400, 600, or 800 nM C646 treatment for 6 h. **B** Protein levels of BmSqstm1, FLAG-BmAtg8, and endogenous BmAtg3 after 100, 200, 400, 600, or 800 nM C646 treatment for 6 h. **C** Immunofluorescent staining of BmAtg3-c-Myc and FLAG-BmAtg8 after 100, 200, 400, 600, or 800 nM C646 treatment for 6 h. Scale bar: 10 micron. **D** Immunofluorescent staining of BmAtg3 and BmAtg8 after 5 μM 20E (6 h), 20 µM TSA (6 h), 5 μM 20E (4 h pretreatment) + 20 µM TSA (2 h in addition), or 20 µM TSA (2 h pretreatment) + 5 μM 20E (4 h in addition) treatment. **E** Protein levels of BmSqstm1, BmAtg3, and BmAtg8 after 5 μM 20E (6 h), 20 µM TSA (6 h), 5 μM 20E (4 h pretreatment) + 20 µM TSA (2 h in addition), or 20 µM TSA (2 h pretreatment) + 5 μM 20E (4 h in addition); 5 μM 20E (6 h), 20 μM CTB (6 h), 5 μM 20E (4 h pretreatment) + 20 μM CTB (2 h in addition) or 20 µM CTB (2 h pretreatment) + 5 μM 20E (4 h in addition); 5 μM 20E (6 h), 800 nM C646 (6 h), 5 μM 20E (4 h pretreatment) + 800 nM C646 (2 h in addition) or 800 nM C646 (2 h pretreatment) + 5 μM 20E (4 h in addition) treatment. Scale bar: 10 micron.

**Fig. S4** Expression and purification of recombinant BmAtg3, BmAtg4, BmAtg7, and BmAtg8. Coomassie blue staining and western blotting of recombinant BmAtg3 (**A**), BmAtg8 (**B**), BmAtg4 (**C**), and BmAtg7 (**D**) with anti-His tag or anti-acetylation antibody. S: supernatant, P: precipitation, Ft: flow-through, W: washing buffer.

**Fig. S5** Acetylation sites identified in BmAtg3, BmAtg8, BmAtg4, and BmAtg7. **A** Two acetylation sites in BmAtg3 identified by mass spectrometry, K: lysine site. **B–B’** Five acetylation sites identified in BmAtg8 (B). Amino acid sequences of Atg8 homologs from *H. sapiens*, *B. mori*, *D. melanogaster*, and *S. cerevisiae* were aligned by DNAMAN software (B’). **C** Three acetylation sites identified in BmAtg4. **D** Seven acetylation sites identified in BmAtg7.

**Fig. S6** Immunofluorescent staining of mutated BmAtg3 and BmAtg8, purification of recombinant HsATG4b, and analysis of its acetylation sites. **A** Immunofluorescent staining of BmAtg3 double acetylation site mutant and BmAtg8 sextuple acetylation site mutant. Scale bar: 10 micron. **B** Coomassie blue staining and western blotting of recombinant HsATG4b detected by anti-His tag and anti-acetylation antibodies. 300 mM-1: 300 mM eluted for 0.5 min; 300 mM-2: 300 mM eluted for 1 min; 300 mM-3: 300 mM eluted for 1.5 min; 300 mM-4: 300 mM eluted for 2 min. **C** Amino acid sequences of BmAtg4 and HsATG4b were aligned by DNAMAN software. Red framework indicates acetylation sites in BmAtg4, and the yellow framework indicates acetylation sites in HsATG4b.

**Fig. S7** Deacetylation of HsATG4b leads to its nuclear export and autophagy. **A** Protein levels of LC3 after the overexpression of *HsAtg4a*, *b*, *c* or *d* in HEK293 cells under nutrient-rich conditions and starvation. **B** Protein levels of SQSTM/p62, HsATG4b, and LC3 after *HsAtg4b* overexpression. **C** Acetylation levels of HsATG4b after starvation for 4 h. **D** Immunofluorescent staining of HsATG4b after starvation for 4 h. **E** Four acetylation sites in HsATG4b were identified by MS analysis. Scale bar: 10 micron. **F** Acetylation levels of HsATG4b-HA after single and quadruple mutation of acetylation sites. 4KR: quadruple acetylation-site mutation from lysine (K) to arginine (R). **G** Immunofluorescent staining of HsATG4b-HA after quadruple mutation of acetylation sites. Scale bar: 10 micron. **H** Protein levels of SQSTM/p62 and LC3B after the overexpression of quadruple acetylation-site mutated *HsAtg4b* under nutrient-rich conditions and starvation. **I** Acetylation of HsATG4b-c-Myc, and protein levels of SQSTM1/p62 after *HsHDAC1* overexpression.

**Fig. S8** Multiple signals regulate autophagy occurrence in insects. Nutrient, energy, and insect molting hormone 20E synergistically regulates autophagy occurrence in insects at both transcriptional and post-translational levels.
